# Cross-sectional United Kingdom surveys demonstrate that owners and veterinary professionals differ in their perceptions of preventive and treatment healthcare needs in ageing dogs

**DOI:** 10.3389/fvets.2024.1358480

**Published:** 2024-04-04

**Authors:** Lisa J. Wallis, Alan D. Radford, Zoe Belshaw, Jodie Jackson, Eniko Kubinyi, Alexander J. German, Carri Westgarth

**Affiliations:** ^1^Institute of Infection, Veterinary and Ecological Sciences, University of Liverpool, Liverpool, United Kingdom; ^2^Department of Ethology, ELTE Eotvos Lorand University, Budapest, Hungary; ^3^EviVet Research Consultancy, Nottingham, United Kingdom; ^4^MTA-ELTE Lendület “Momentum” Companion Animal Research Group, ELTE Eotvos Lorand University, Budapest, Hungary; ^5^Institute of Life Course and Medical Sciences, University of Liverpool, Liverpool, United Kingdom

**Keywords:** dogs, veterinarians, attitude, communication, delivery of healthcare

## Abstract

Perceptions of dog owners and veterinary professionals (surgeons/nurses) can influence the preventive healthcare and treatment provided to dogs, especially at the senior life-stage, when chronic diseases become more common. This study compared perceptions of healthcare of dogs as they age between dog owners and veterinary professionals. Data from two online surveys (owners: *N* = 633, veterinary professionals: *N* = 305) examined perceived need for veterinary visits and vaccinations. In addition, 48 clinical signs were rated on perceived prevalence (whether owners’ dogs had experienced them) and how urgently they should seek veterinary advice. Groups were compared using descriptive statistics and chi-square. Owners most often believed a ‘healthy’ senior dog (>7 years) should go to the vet once a year (47% owners vs. 25% veterinary professionals, *p* < 0.001), compared with veterinary professionals every 6 months (39 vs. 73%). A minority (14%) of owners would just take the dog ‘if they got sick’ but only 2% of veterinary professionals advised this, and 16% of owners of dogs of all ages had not had any contact with their veterinary practise in the previous year. Nearly all veterinary professionals (92%) believed that senior/geriatric dogs should receive yearly vaccinations. However, 28% of owners’ dogs of all ages were not vaccinated in the previous year and, of these, 33% did not believe that older dogs need vaccinations. Only 10% of dogs considered ‘old’ by their owners had attended a senior wellness clinic or examination, despite 14% of practises offering them. The three most common clinical signs reported by owners were slowing down on walks (57%), dental tartar (53%) and being stiff on rising (50%). Owners perceived urgency to seek veterinary care was lower if they had experienced the clinical sign before. In the current study, dog owners and veterinary professionals differed in their opinions about the need for veterinary care, suggesting new educational initiatives, and more effective communication is required.

## 1 Introduction

Lifespan and similarly the age at which a dog is classified as senior in scientific literature varies according to size, weight, breed, lifestyle, and health status (1). The senior life stage has been defined as the last 25% of a dog’s estimated lifespan through to the end of life (2). Previous research has determined that most owners begin to view their dogs as senior (or old) from the age of 6 years onwards, which likely corresponds to the beginnings of cognitive (3, 4), sensory and physical decline (5). Lack of healthcare provision for senior dogs is a significant welfare issue (6) especially in light of the ageing dog population (7). Since senior animals make up 30–40% of the animals owned by the veterinary client base, it is argued that implementing a successful senior health care programme is imperative to provide long-term health and patient wellbeing (8, 9). Traditionally, veterinarians use annual vaccination appointments as their main opportunity to assess the health of older pets under their care. However, 23% of dogs do not receive regular yearly boosters (10) and, of those that do, the focus in consultations is normally on current physical health problems, with only a limited discussion of preventive healthcare due to time constraints. These appointments also often lack a standardised structure or educational focus (11).

Since over 4.2 million dogs in the United Kingdom are not insured (44% of the United Kingdom dog population) (estimated to be 9.6 million in 2021) (10), mainly due to high prices (12) and insurance plans for senior and geriatric dogs tend to be more expensive, it is likely that many receive lower levels of healthcare from a veterinary professional than ideal. Further, when apparently healthy (according to the owners) dogs aged above 9 years were screened for age-related issues, veterinary surgeons identified at least one previously unrecognised problem in 80% of them (13, 14).

Previously, we determined that canine cognitive dysfunction (CCD) was a priority concern for owners but not for veterinary professionals (15). Given that behavioural changes associated with age-related CCD are common, and there is an exponential increase in CCD prevalence with increasing age (with up to 23 per cent of dogs >9 years being affected), screening for age-related behavioural changes is important (16, 17). However, owners often do not recognise or voluntarily report clinical signs especially if they are mild (18). This may be because they consider them to be a normal part of the ageing process and do not understand the significance of the signs or the importance of early identification and intervention (15, 19), or they are reluctant to attend an appointment with an apparently ‘healthy’ senior pet (20), due to finances, inconvenience, or potential stress to the owner and the dog. Unless senior and geriatric dogs receive regular health and behavioural screening, including any necessary treatment and follow up, their long-term health and wellbeing cannot be assured, as a change in behaviour may not only be indicative of CCD but might be the first or only sign of disease, pain, and impaired welfare. In the first qualitative description of United Kingdom senior dog healthcare from the point of view of both owners and veterinary professionals, opportunities for educating owners on what behavioural and physical signs represent normal or ‘healthy’ ageing were identified, as well as risks of missing occult disease due to lack of time, education and, in some cases, motivation (15). Further, in contrast to the United States where the American Animals Hospitals Association (AAHA) have issued both canine life-stage and senior care guidelines (2, 21), there are no such guidelines currently in the United Kingdom.

Senior wellness clinics, screening, or examinations have been trialled in some veterinary practises in the United Kingdom, whereby veterinary professionals focus on problems specific to ageing, typically performing a complete physical examination and clinicopathological assessments (e.g., blood tests and urinalysis), in order to detect occult disease at an early stage or to monitor stable ongoing health problems (20). Senior wellness clinics are distinct from annual wellness exams, in that they focus on problems specific to ageing, and occur regardless of health status, whereas wellness exams are recommended yearly for adult dogs, and are typically performed on dogs that appear healthy. Pet health plans, which typically include vaccines, parasiticides, free consultations and free reminder alerts for owners, are also increasing in popularity (22), and can include a senior health plan option. However, it is well known that owners can be reluctant to pay the additional costs of diagnostic tests and practises are limited in time to implement them (8).

Belshaw et al. (11) called for an expansion of the definition of preventive healthcare to include routine screenings, check-ups, and client counselling about nutrition, behavioural issues and chronic diseases (such as obesity and dental disease). Senior dog healthcare plans, wellness clinics and adequate insurance coverage could all support a programme of healthcare for elderly dogs. However, to date, no studies have evaluated the prevalence of these approaches in veterinary consultations with senior pets in the United Kingdom. Research into the demographics of the senior dog population is lacking, including health conditions and clinical signs recognised by owners, and their decision-making around urgency to seek care or what signs they attribute to old age rather than disease. Therefore, the aim of this study was to use online questionnaires to compare the perspectives of dog owners and veterinary professionals on the health and preventative care needs of dogs as they age. We also aimed to determine how senior dog preventive healthcare or treatment advice is currently offered in United Kingdom veterinary practises, what health conditions and clinical signs are of primary concern to both owners and veterinary professionals, and whether a pre-appointment owner questionnaire to help identify potential problems would be welcomed by owners and veterinary professionals.

## 2 Methods

Descriptive cross-sectional surveys of dog owners and veterinary professionals were utilised to measure the frequency of dog health conditions, clinical signs, urgency to seek care, perspectives of preventive care needs, and current and potential veterinary senior dog practise tools used or for use by veterinary professionals and owners. The questionnaires were designed in a three-stage process, where firstly semi-structured interviews were conducted with dog owners and veterinary professionals to determine (1) how senior dog preventive health care or treatment advice is currently offered in United Kingdom veterinary practises; (2) health conditions and clinical signs that are primary concerns to owners and veterinary professionals; and (3) barriers to delivery of senior dog health care and ideas for best-practise solutions. The results of the thematic analysis can be found in Wallis et al. (15). Secondly, common health conditions and clinical signs mentioned in clinical narratives in first opinion veterinary electronic health records were reported in a separate publication (23), and used to help construct the questionnaires. Once draft versions of the questionnaires were completed based on these, piloting of the questionnaires was conducted in a small sample of dog owners and veterinary professionals (*N* = 10 each), and the study research team and participants were consulted to perfect the design. Some of the results of the final questionnaires are not reported here, such as the caregiver burden and end of life questions, as these will be presented in a separate future publication. Due to the descriptive nature of the study and the large number of questions explored, some of the results are presented in the Supplementary material A.

### 2.1 Questionnaire recruitment

Participants were recruited through advertisements on social media, press releases, and emails to the British Small Animal Veterinary Association (BSAVA) members. As an incentive, a prize draw for a £50 voucher from a well-known multinational company was offered to dog owners and a separate voucher to veterinary professionals who completed the survey. Incentives were offered as they are known to increase participation by up to 19% on average, and improve completion rates by up to 27% (24). Online surveys allow participants to misrepresent their eligibility to participate or mask their identity in order to participate multiple times (25), and offering incentives may increase fraudulent activity (26). Due to this reason, the data underwent additional scrutiny to identify possible ‘fake’ respondents, who provided responses in order to complete the questionnaire, but did not meet the eligibility requirements (27). Eligibility criteria for dog owners included being aged 18 or older, living in the United Kingdom, and the current or recent possession (within the last 12 months) of a dog of any age. The data provided by owners of dogs of all ages were included in the analyses as their views and opinions on senior dog healthcare and decisions regarding the necessity of veterinary visits for experienced and hypothetical clinical signs were valid regardless of the age of their current dog. If owners had more than one dog, they were asked to complete the survey for the oldest dog that they owned. If the oldest dog had died in the past year, they were asked to complete the questions for that dog, thinking about the last 3 months of life. The questionnaire was open from 09/09/2021 to 18/11/2021.

For veterinary professionals, eligibility criteria were being aged ≥18, living and practising in the United Kingdom, and the current or recent (within the past 2 years) participation in consultations on senior dog preventive healthcare. Eligible professionals included veterinary surgeons, veterinary nurses, and veterinary physiotherapists. The questionnaire was open from 12/10/2021 to 25/12/2021.

Decisions on whether to exclude participant submissions from the datasets were made according to the Reflect, Expect, Analyze, and Label Framework (REAL) (27); for example, where participants filled in incorrect/inconsistent qualifications (veterinary professional questionnaire) or demographic information, answered open questions in a language other than English or with nonsense words, clicked the first response on each possible multiple-choice answer (for multiple questions), and/or that followed a set pattern of responses which did not deviate. Duplicates were removed where entries were made close together and did not differ in their answers, or if the timestamp showed many entries on the same day/time, with a short duration to completion, with multiple anomalies (most often including incorrect/inconsistent qualifications for the veterinary professional questionnaire).

#### 2.1.1 Dog owner questionnaire

A copy of the questionnaire can be found in the Supplementary material B. In brief, the owner questionnaire comprised an introductory text and informed consent section, dog demographics, attitudes to dog healthcare and wellness checks, health conditions, clinical signs, owner demographics, and an end of survey debrief.

Owner or family unit variables included owner sex, age, education, and income as well as the number of dogs living in household. Demographic variables of the dog included age (in years), sex, neuter status, size (toy, small, medium, large, and giant), current weight (in kg), owner perceived body condition score (BCS; Supplementary material B) as well as whether the dog had died, would be described as old, their insurance status and whether the dog was receiving medication (Supplementary Table 1). Dogs were assigned to three breed groups: cross breeds (where the parents were known purebreds of different breeds), purebreds (parents were of one known breed/pedigree), and mixed breeds (where the parents of the dog were of unknown breed status) (Table 1).

**Table 1:** Descriptive statistics of the dogs, including sex, age in years, weight in kg, and body condition score (range from 1 to 9) displayed by breed group (crossbreed, pure bred, and mixed breed).

		Sex *N* (%)		Age in years, median (IQR) *N* = 582	Weight in kg, median (IQR) *N* = 548	Body condition score, median (IQR) *N* = 575
Breed	Total count (%)	Male	Female			
Crossbreed	112 (19.5)	60 (53.6)	52 (46.4)	9 (0.5–17.5)	18 (4.0–32.0)	5 (4.0–6.0)
Purebred	363 (63.2)	185 (51.0)	178 (49.0)	11 (5.0–17.0)	20 (2.0–38.0)	5 (4.0–6.0)
Mixed breed	99 (17.3)	40 (40.4)	59 (59.6)	13 (7.0–19.0)	17.5 (4.5–30.5)	5 (3.0–7.0)
Chi Squared/ Kruskal Wallis		Chi-squared = 4.325, *p* = 0.115		T = 24.007, df = 2, ***p* < 0.001**	T = 8.313, df = 2, ***p* = 0.016**	T = 0.028, df = 2, *p* = 0.986
Grand total	574	285	289	11 (5.0–17.0) *N* = 630	19 (2.0–36.0) *N* = 595	5 (4.0–6.0) *N* = 623

IQR, Interquartile range; T, *t* value, df, Degrees of freedom; *p*, *p* value. A Chi-squared test was run to examine whether the proportion of males and females differed between the three breed groups, and Kruskal Wallis tests were conducted to look for median group differences in age, weight, and body condition score.

Owners were also asked about their dog’s vaccination status in the last year, including primary vaccinations or boosters for Distemper, Parvovirus and infectious canine hepatitis virus, or yearly boosters for leptospirosis and kennel cough. The British Small Animals Veterinary Association (BSAVA) recommends that in the United Kingdom, core vaccines for dogs include Leptospirosis, which must be administered annually (28). If owners reported that the dog was unvaccinated, they were asked about why this was. Owners were asked to select from a list of 20 health conditions (adapted from common conditions diagnosed in senior pets) (29) and indicate any that their dog had suffered over their lifespan and whether they had received a formal veterinary diagnosis, were diagnosed by someone other than a veterinary surgeon, or the diagnosis had been assumed by the owner based on the presence of compatible clinical signs. Owners were also asked whether they had ever observed each of 48 clinical signs in their dog, if so, at what age, and whether they had/would seek veterinary advice for each (urgency to seek care; six-point Likert scale). Owners who would not seek veterinary advice were asked whether they believed the sign was a normal part of ageing. Owners also reported how often they had visited their veterinary practise in the past year with their oldest dog, whether their dog was enrolled in a health plan, had attended a senior dog wellness clinic or examination, and were also asked about the design of a possible future senior dog healthcare education resource. Finally, they were asked how often they believed a senior dog should visit a veterinary professional if they appeared to be healthy.

#### 2.1.2 Veterinary professional questionnaire

A copy of the veterinary professional questionnaire can be found in the Supplementary material C. In brief, the questionnaire comprised: an introductory text and informed consent, participant’s profession and practise demographics (including whether senior health checks or plans, and wellness examinations or clinics were undertaken), their personal views on senior dog healthcare and clinical signs, a personal demographic section, and, finally, an end-of-survey debrief.

Veterinary professional demographic variables included profession, sex, age, ethnicity, length of service, practise category, employment type, and practise type (Supplementary Table 2). Veterinary professionals were asked whether their practise was currently conducting routine health checks, offering health plans, or senior dog wellness clinics, and if not, why. They were asked at what age they would expect a typical medium-sized dog to be senior, whether a different approach to consultation is required for senior dogs, how often senior dogs should visit a veterinary practise if they appear to the owner to be healthy, whether they should continue to receive annual booster vaccinations (and, if not, why), and also questions about the design of a possible future senior dog healthcare education resource. Similarly, veterinary professionals were asked how important they thought it was for owners to seek veterinary advice for the same 48 clinical signs as the owner questionnaire, and how often (on a seven-point Likert scale) they thought owners attribute the different clinical signs to ‘just old age’ and not seek veterinary advice.

### 2.2 Data analysis

Given that this was an exploratory study, results are mainly presented as descriptive statistics and univariate analyses [Statistical Package for the Social Sciences (SPSS) version 22, SPSS Inc.; R version 4.0.2, The R Foundation for Statistical Computing, http://www.R-project.org, and the package ‘likert’] (30). This included median and interquartile range (IQR) for continuous data or number (%) for categorical data. Non-parametric statistical tests were used to compare groups (between veterinary surgeons and veterinary nurses and, where possible, between dog owners and veterinary professionals). For continuous variables (e.g., dog age and body weight) either the Kruskal-Wallis (*post-hoc* Dunn-Bonferroni tests on each pair of groups) or Mann–Whitney test was used, and for categorical data Chi-Square tests were used, with Chi-square tests with a Bonferroni correction for *post-hoc* testing. Comparisons between dog owners and veterinary professionals were conducted on perceptions about senior dog healthcare and wellness checks, urgency to seek care, attribution of clinical signs to old age, and future senior dog healthcare resource design. The significance level for all tests was set at *p* < 0.001 to help control for Type I errors, due to the large number of comparisons.

An *a-priori* calculation of sample size using the programme GPower (31), showed that 372 participants would be needed for a Chi Squared goodness of fit test with 5 degrees of freedom sensitive enough to detect a significant difference between groups with a medium effect size of Cohen’s *w* = 0.3 (*α* = 0.001, power = 0.95).

## 3 Results

### 3.1 Questionnaire responses

Of 1,152 dog owner participants, 445 entries were removed as incomplete to the end of the survey, 45 as not living in the United Kingdom, 11 because the participant did not currently own a dog (or had not done so within the last year), and 18 as duplicates, non-English or ‘fake’ respondents. The final cleaned sample comprised 633 dog owners (55% of the original 1,152 dog owner participants).

Of 696 veterinary professional respondents, 220 entries were removed as incomplete, 22 were not living in the United Kingdom, 27 did not currently work as a veterinary professional (or had not within the last 2 years), and 122 were either duplicates, contained errors or were from ‘fake’ respondents that completed the survey in order to enter the incentive prize draw. The final cleaned sample comprised 305 veterinary professionals (44% of the original 696 veterinary professional participants).

### 3.2 Demographics

Dog, owner, and veterinary professional demographics are shown in Supplementary Tables 1, 2. Dogs median age in years was 11 (IQR 5–17, range 3 months–20 years), and mixed breeds were older than purebred and cross-bred dogs (Table 1). 162 dogs (26%) had passed away, at a median age of 13 years (IQR 11–15). Three hundred and thirty-six dogs (53%) had medical insurance, and dogs that were not insured were older than dogs that were insured [12 years (IQR 9.5–14.5) vs. 9 years (IQR 5.5–12.5), *p* < 0.001]. Three hundred and ten dogs (49%) were receiving medication prescribed by a veterinary surgeon, and these were older than those who did not receive medication [12 years (IQR 10–14) vs. 8 years (IQR 4–12), *p* < 0.001]. Five hundred and thirty-one dogs (84%) were neutered, and these dogs were older (11 years, IQR 8.5–13.5) than those reported to be sexually intact (8 years, IQR 3.3–12.3) (*p* < 0.001).

Owners were predominately female (584, 92%), with a degree (183, 29%) or a higher degree (158, 25%) and owner age and income was spread equally across categories (Supplementary Table 1). Eighty percent of veterinary professionals were veterinary surgeons (245), and the rest were veterinary nurses (60, 20%). Only two veterinary physiotherapists completed the questionnaire, and these were both veterinary nurses, and so they were classified as veterinary nurses. Veterinary professionals were predominately female (255, 84%), aged between 30 and 40 years (81, 27%), and were employed at a small animal (268, 88%) and corporate practise (158, 48%) (Supplementary Table 2).

### 3.3 Perceptions about senior dog healthcare and wellness checks

Owners who thought their dog had reached old age (384, 61%, median dog age: 13 years) believed that this had happened at a median age of 11 years (IQR 9.5–12.5) [toy/small 11 years (IQR 8–14), medium 11 years (IQR 8–14), and large/giant 10 years (IQR 7–13), *p* < 0.001]. Veterinary professionals considered a medium-sized dog (such as an English Cocker Spaniel) to be ‘senior’ at a median age of 8 years (IQR 7–9). Owners (290, 47%) most often believed that a ‘healthy’ senior dog (defined to them as >7 years of age) should visit their veterinary practise once per year, whilst 89 (14%) stated that they would only take the dog if they got sick; in contrast, veterinary professionals favoured more frequent visits [e.g., 73% (219) indicated every 6 months, *p* < 0.001; Table 2].

**Table 2:** Results of the Chi-squared analysis of dog owner and veterinary professional opinion on how often a senior dog should go to the vet if they seem healthy.

Question	Categories	Dog owner (DO) (*N*, %)	Veterinary professional (VP) (*N*, %)	Veterinary surgeon (VS) (*N*, %)	Veterinary nurse (VN) (*N*, %)	Chi-square (*p*)
How often should a senior dog go to the vet if they seem healthy?	Every 6 months	244 (39)	219 (73)	177 (73)	42 (72)	0.0, (0.981) VS VN, 39.6, (**<0.001**) DO VP
	Every year	290 (47)	76 (25)	61 (25)	15 (26)	
	Only if they got sick/owner led	89 (14)	6 (2)	5 (2)	1 (2)	
	Missing	10	4	2	2	

If veterinary surgeons and veterinary nurses did not differ, owners were compared with all veterinary professionals. The category missing was not included in the analyses. DO – dog owner, VP – veterinary professional, VS – veterinary surgeon, VN – veterinary nurse.

**Figure 1:**
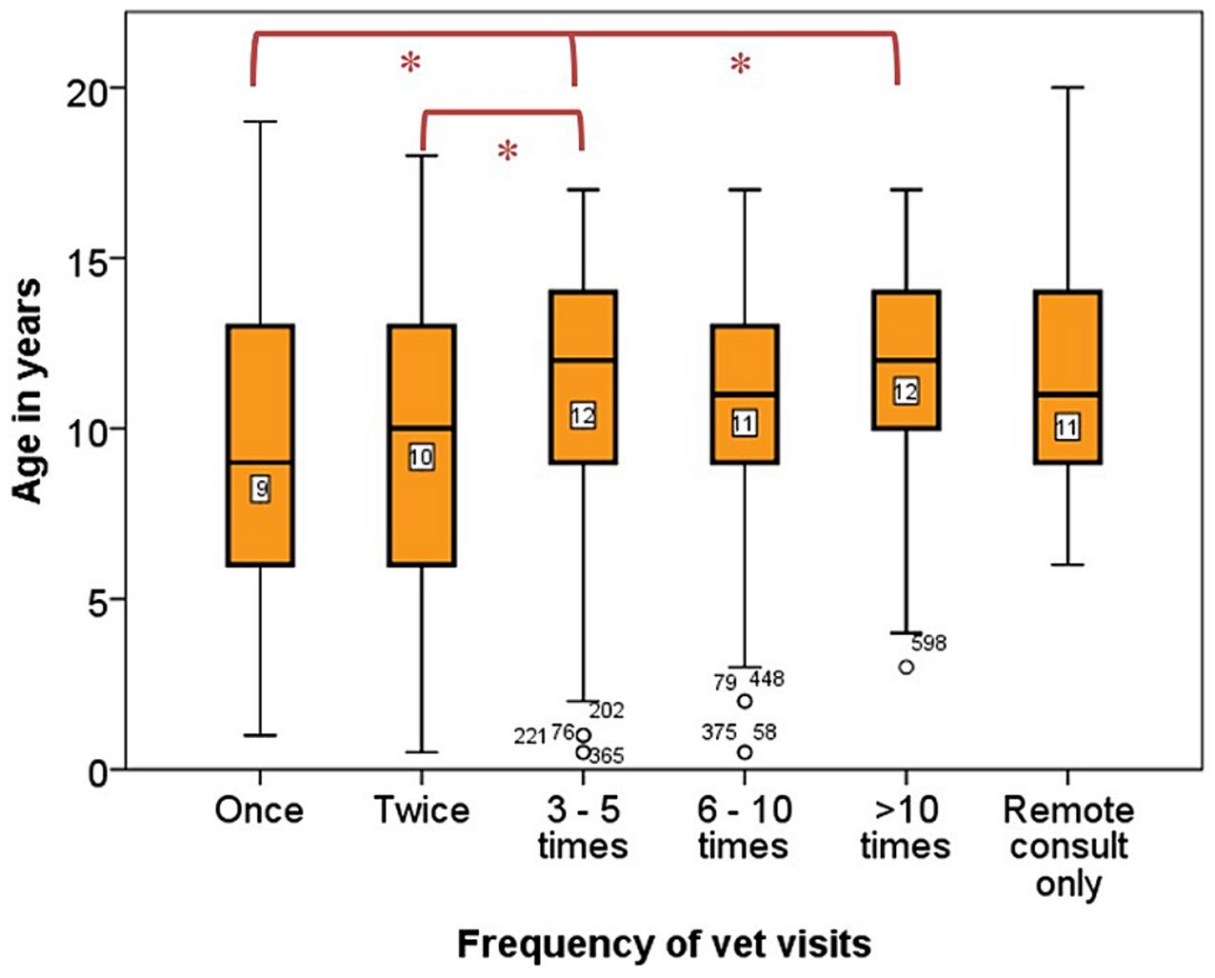
Box plot displaying frequency of times owners brought their dog physically to the veterinary surgeon in the past 12 months and age in years of the dogs. Number of dogs = 503. The frequency of vet visits was categorised into five groups: once, twice, 3–5 times, 6–10 times, >10 times and remote consult only. The median age in years of the dogs in each frequency category were compared using a Kruskal Wallis test, df = 5, *N* = 503, *p* = 0.001. Categories which differed significantly are denoted with ^*^p < 0.050, significance values have been adjusted by the Bonferroni correction for multiple tests.

Owners of dogs of all ages most often presented their dog to the veterinary surgeon 3–5 times per year (171, 33%), with a small proportion visiting more than 10 times (43, 8%) (See Supplementary Table 3). One hundred and four owners (16%) had not consulted with a veterinary surgeon in the past 12 months, and these dogs were younger (median 9 years, IQR 2–16) than dogs whose owners had consulted their veterinary surgeon (11 years, IQR 6–16; *p* < 0.001). Likewise, older dogs visited the veterinary surgeon more frequently (Figure 1; *p* = 0.001). The most frequent reason for a veterinary visit was a routine health appointment (352, 44%) which could include for example a vaccination, anal gland check, nail clip, or medication recheck, followed by a new health condition or illness (288, 36%), and then advice on euthanasia and end of life care (87, 11%).

According to veterinary surgeons, most practises did not have a standardised consultation protocol during annual booster appointments or for new patients (Table 3), and veterinary surgeons were more likely to have developed their own informal protocol compared with veterinary nurses [136 (59%) vs. 6 (11%)]. Most veterinary professionals (260, 85%) believed that senior dogs require a different consultation approach than younger dogs; however, their practise did not standardise this (261, 89%), and 110 (38%) veterinary professionals had developed their own (Table 3).

**Table 3:** Results of the Chi-squared analysis to determine whether veterinary surgeons and veterinary nurses differed in their opinions of veterinary professional questions examining standardised consults, health plans, senior wellness clinics, and own beliefs concerning the healthcare of senior dogs.

Question	Categories	Veterinary professional (*N*, %)	Veterinary surgeon (*N*, %)	Veterinary nurse (*N*, %)	Chi-square (*p*)
Does your practise have a standardised consult on dog healthcare?	No, but I personally have my own which I carry out regardless of age	142 (50)	136 (59)	6 (11)	39.6 (**<0.001**)
	Yes, all animals receive a standardised full health check regardless of age	144 (50)	96 (41)	48 (89)	
	Missing	19	13	6	
Do you think senior dogs require a different consultation approach than with younger dogs?	No, all consultations should follow the same procedure regardless of age	41 (14)	37 (15)	4 (7)	5.5 (0.064)
	Yes, but I do not currently do this	44 (15)	31 (13)	13 (23)	
	Yes, and I already do this	216 (71)	176 (72)	40 (70)	
	Missing	4	1	3	
Does your practise have a standardised consult on senior/ geriatric dog healthcare	No	151 (51)	124 (51)	27 (51)	21.6 (**<0.001**)
	Not formally offered, but perform own checks	110 (38)	99 (41)	11 (21)	
	Yes	33 (11)	18 (8)	15 (28)	
	Missing	11	4	7	
Does your practise offer health plan/s to owners?	No, and not interested in offering them	29 (11)	25 (11)	4 (9)	2.2 (0.337)
	No, but I would be interested in us offering them	17 (6)	12 (5)	5 (11)	
	Yes, we offer at least one health plan	222 (83)	186 (84)	36 (80)	
	Missing	37	22	15	
Does your practise offer a health plan specifically for senior dogs?	Yes	43 (19)	35 (19)	8 (22)	0.2 (0.636)
	No/generic plan for all life stages	179 (81)	151 (81)	28 (78)	
Does your practise offer senior dog wellness clinic/examinations?	No	180 (67)	152 (68)	28 (61)	5.2 (0.155)
	Not currently, but ran one previously	45 (17)	36 (16)	9 (19)	
	Yes	39 (14)	33 (15)	6 (13)	
	Do not know	6 (2)	3 (1)	3 (7)	
	Missing	35	21	14	
Do you believe that senior and geriatric dogs should receive annual booster vaccinations?	No	24 (8)	19 (8)	5 (8)	0.0 (0.902)
	Yes	278 (92)	223 (92)	55 (92)	
	Missing	3	3	0	

The category missing was not included in the analyses.

Two hundred and twenty-two veterinary professional (83%) worked at practises, which offered healthcare plans to owners, although of these, only 43 (19%) had a plan specifically for senior and geriatric dogs (Table 3). Finally, 39 (14%) of the practises offered senior dog wellness clinics or examinations, although 45 (17%) ran one previously but stopped due to lack of time, space, or personnel, poor client uptake, and COVID. These were managed more often by veterinary nurses (22, 56%) than veterinary surgeons (14, 36%) but some (3, 8%) were run by both. They typically included: full clinical examination, full blood profile, routine urinalysis, and blood pressure measurements, with some discussion of diet, dental care, exercise, home environment, and management of chronic diseases (such as osteoarthritis). The main reasons given why such clinics were not run (*N* = 158) were (multiple selections were possible): not enough time (85, 54%), personnel (74, 47%), space (44, 28%), and poor client uptake due to increased costs of diagnostics (43, 27%), but 58 (37%) hoped to offer them in the future.

One hundred and sixty-one dogs (25%) from the owner questionnaire were signed up to a veterinary health plan (Supplementary Table 3) and these dogs were younger than those not on a health plan (9 years, IQR 1–17, vs. 11 years, IQR 6–16, *p* < 0.001). Forty-six dogs (7%) of all ages from the full sample had attended a senior dog wellness clinic or examination (10% of dogs considered ‘old’ by their owners; Supplementary Table 3). Of the 280 dogs considered to be old by their owner, that had not attended (median age 12, IQR 8–16), 80 owners (29%) stated that it was not offered, and the remaining 200 owners (71%) did not know whether it was offered. Two hundred and fifty-one owners of dogs of any age (43%) who had not attended a senior dog wellness clinic or examination said they might attend one if offered, and 115 (20%) replied only if it was free.

One hundred and seventy-five dogs of any age (28% of the full sample) had not received a vaccination in the past 12 months (Supplementary Table 3), and dogs which were not vaccinated were older than those that were (median age 12 years, IQR 7–17, compared with 10 years, IQR 3–17, *p* < 0.001). When asked why their dog was not vaccinated, 57 owners (33%) replied that older dogs do not need vaccinations, 50 dogs (29%) received puppy vaccinations only, and 29 owners (17%) titre test and only vaccinate if needed (Supplementary Table 3). In contrast, nearly all veterinary professionals (278, 92%) believed that senior and geriatric dogs should continue to receive annual booster vaccinations (Table 3), whilst reasons for believing annual vaccinations should not be given included the presence of an underlying health condition, the belief that a lifetime of boosters likely meant good immunity, and dog lifestyle (e.g., no longer meeting other dogs). In such cases, the median age stated from which they would stop routinely vaccinating dogs was 10 years (IQR 6.2–13.8, *N* = 20).

### 3.4 Health conditions and clinical signs

**Figure 2:**
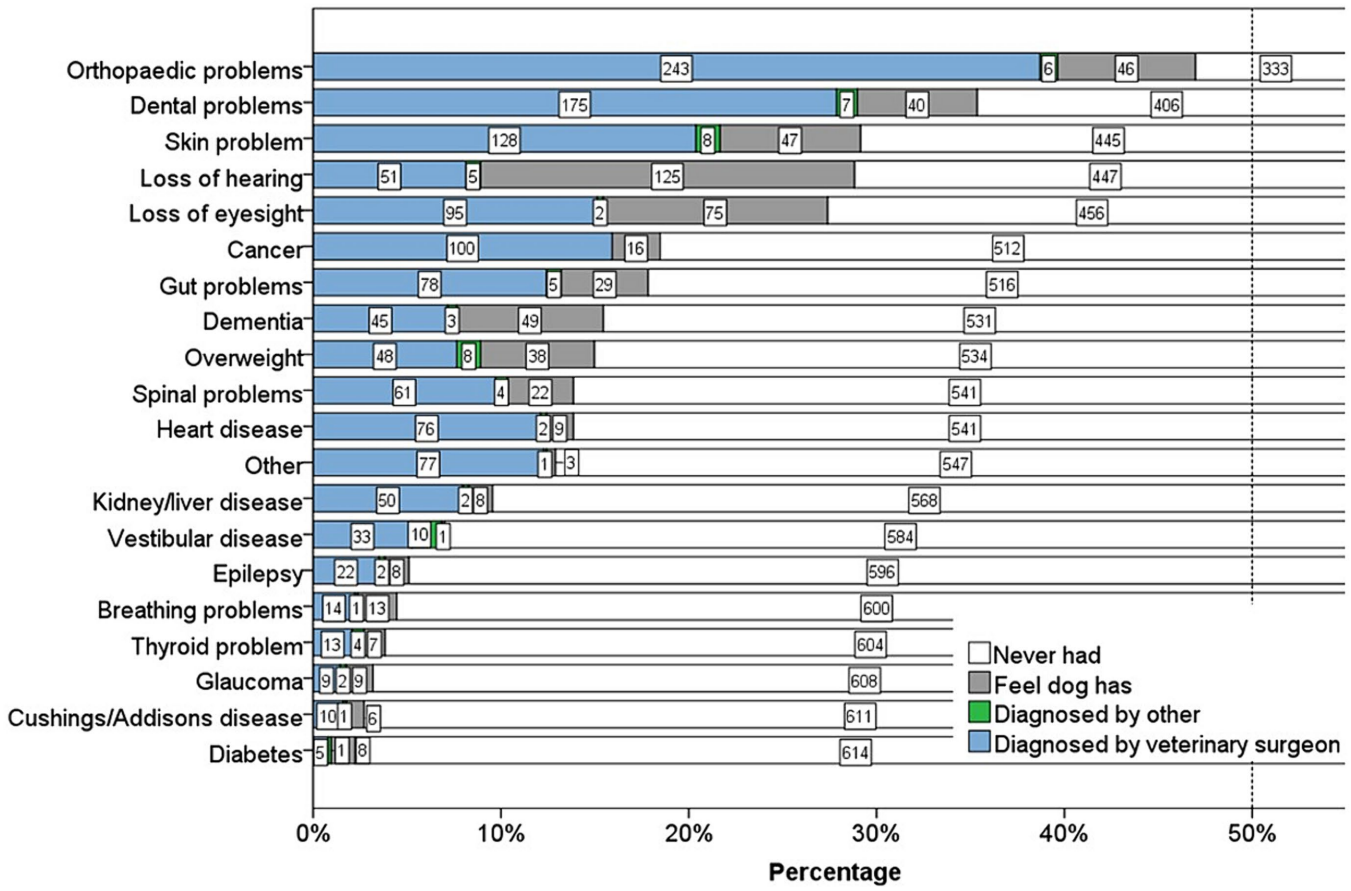
Stacked bar chart displaying the percentage of dogs who suffered from 20 different health conditions, and how they were diagnosed. Number of dogs = 628. Diagnosis was categorised into four groups: diagnosed by a veterinary surgeon, by someone other than a veterinary surgeon, the dog had not been formally diagnosed but the owner felt their dog had it, or the dog had never had the health condition. The numbers in the bars are counts of dogs in each category of diagnosis. The dotted line represents 50% of the sample.

The most common health conditions reported by owners from the list of 20 included in the dog owner questionnaire were musculoskeletal (orthopaedic problems: 295, 47%), dental (dental problems: 222, 35%), integument (skin problems:183, 29%), sensory loss (hearing: 181, 29%, eyesight: 172, 27%), neoplasia (cancer: 116, 19%), digestive (gut problems: 112, 18%), and neurological [dementia (CCD): 97, 15%]. For some health conditions including loss of hearing, dementia, breathing problems and glaucoma, 50% or more of the dogs that had issues had not been officially diagnosed by a veterinary surgeon (Figure 2).

**Figure 3:**
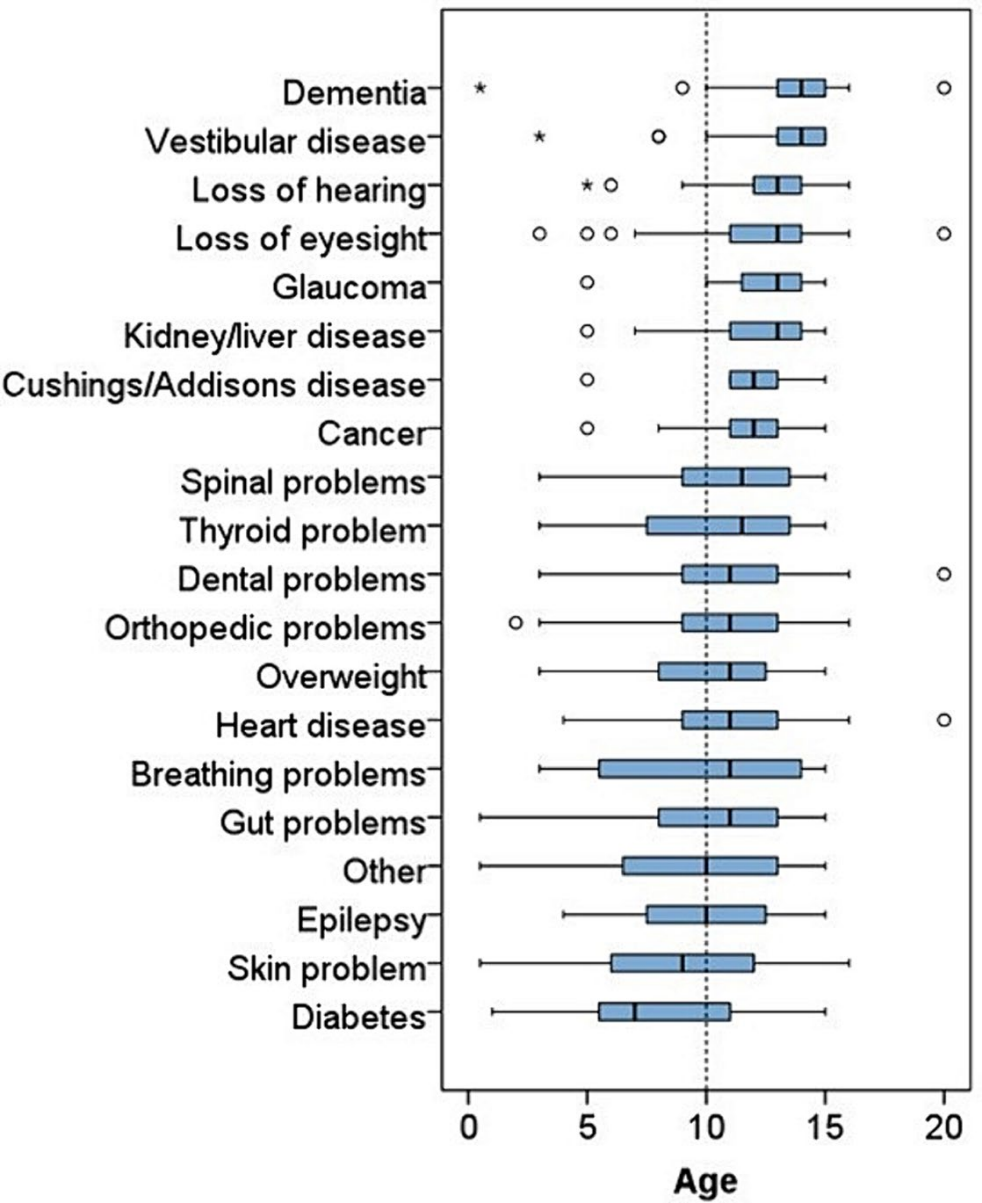
Stacked box plot displaying the age in years of the dogs that suffered from the 20 different health conditions.

Some health issues were more likely to affect middle-aged and senior dogs (diabetes: median age 7, skin problems: 9 years, epilepsy: 10 years and other: 10 years), whilst other conditions affected older dogs (dementia: median age 14, vestibular disease: 14 years, loss of hearing and eyesight: 13 years, glaucoma: 13 years, and kidney/liver disease: 13 years) (Figure 3).

**Figure 4:**
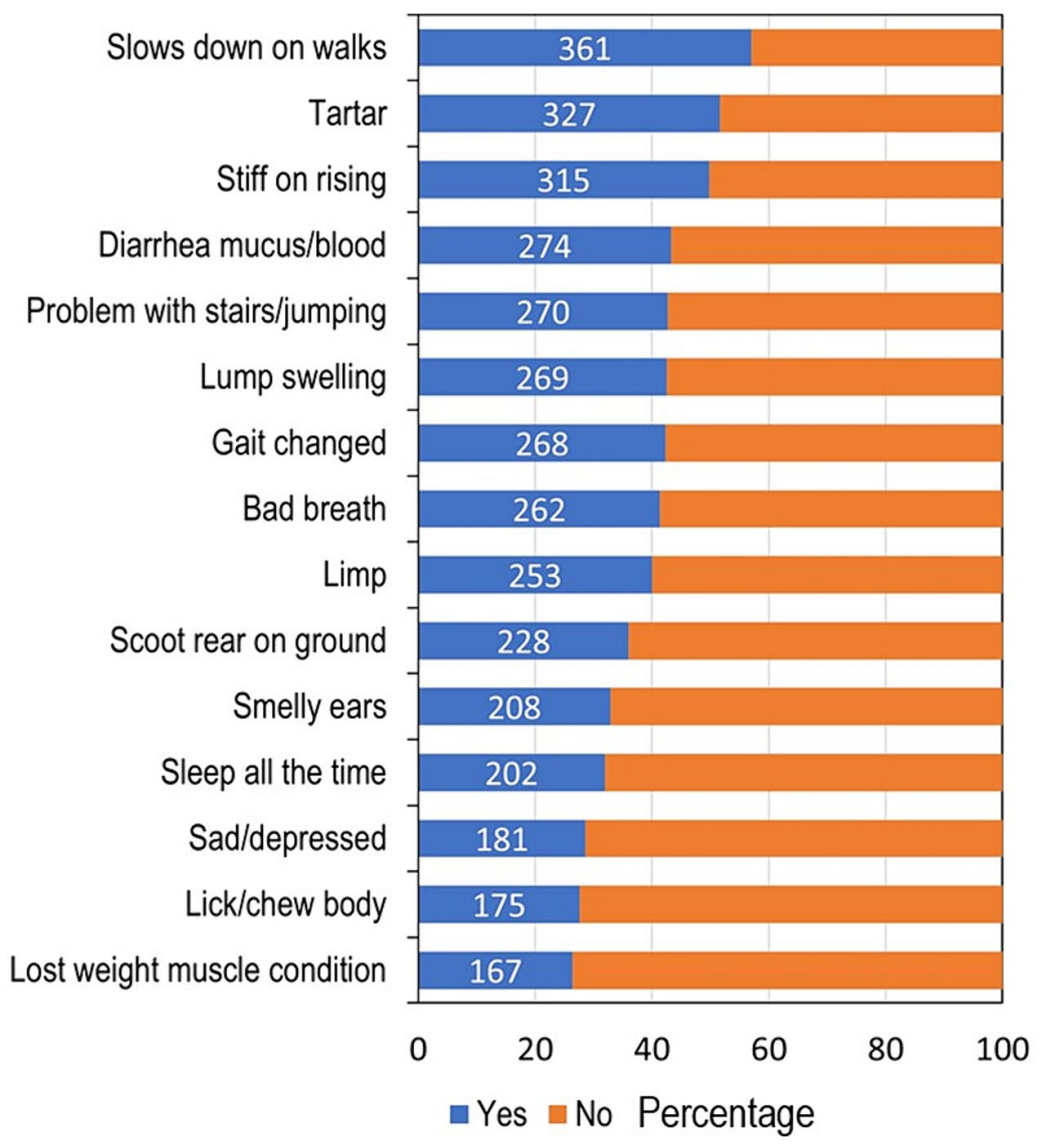
Stacked bar chart displaying the percentage of dogs who suffered from the 15 most common clinical signs. The clinical signs are listed in order of increasing prevalence. Blue bars indicate the percentage of dogs whose owners reported they had experienced that clinical sign, with the actual frequency of dogs written in numbers; the orange bars indicate the percentage of dogs, which had not experiences the clinical sign.

**Figure 5:**
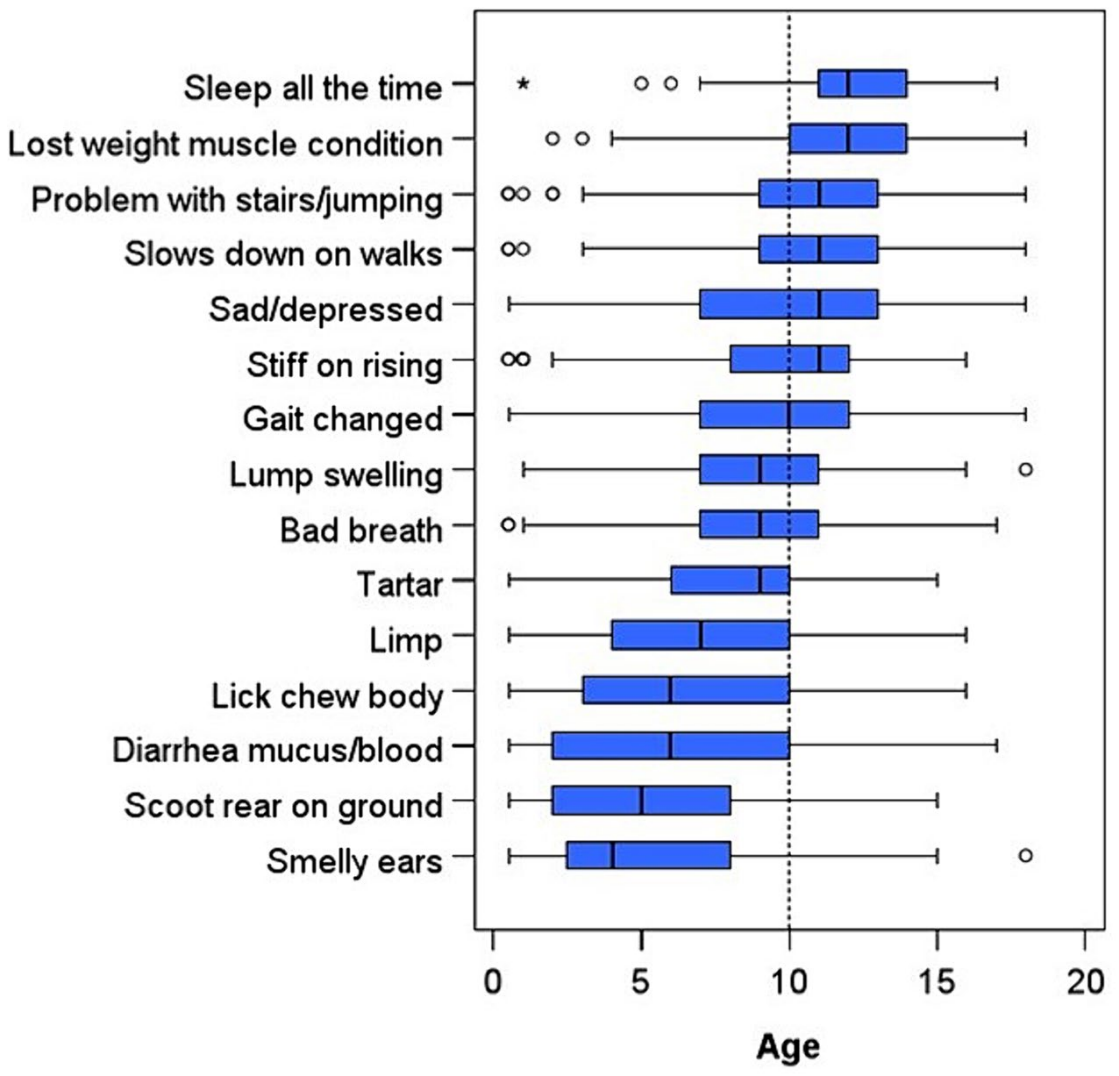
Stacked box plot displaying the age in years each dog was reported by their owner to have first experienced each of the 15 most common clinical signs. The clinical signs are listed in order of increasing age of onset.

Out of the 48 clinical signs, slowing down on walks was the most common clinical sign observed by all owners in the dog owner questionnaire (361, 57%, Figure 4) and most often occurred in dogs between 9 and 13 years of age (Figure 5). Calculus (tartar) was the second most common issue (327, 52%) and was observed from 6 to 10 years of age; results for halitosis (bad breath: 262, 42%) were similar. Two hundred and sixty-nine dogs (43%) had experienced a lump or swelling in the skin, and the median age this occurred was 9 (IQR 7–11). Two hundred and two dogs (32%) were described as spending nearly all their time sleeping, and this sign first occurred most often between 11 and 14 years of age. One hundred and eighty-one dogs (29%) had experienced being sad, lethargic, depressed or disinterested in life, which commenced around 7 years of age.

### 3.5 Urgency to seek care

**Figure 6:**
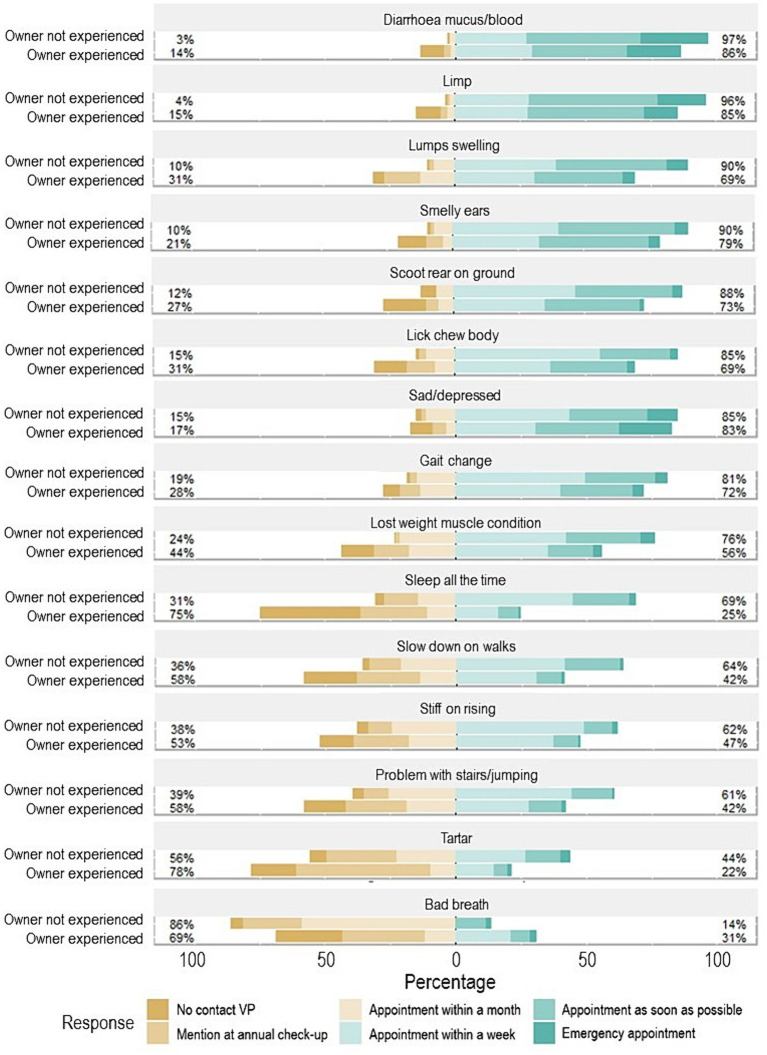
Diverging stacked bar chart showing urgency to seek care for the 15 most common clinical signs. Urgency to seek care was categorised into a six-point Likert scale divided into lower and higher urgency including: no contact with a veterinary professional, mention at annual check-up, and appointment within a month (which are all depicted in shades of brown and represent the lower urgency to seek care categories, on the left-hand side of the *x* axis of percentage of owners that reported on each clinical sign), and the categories appointment within a week, appointment as soon as possible, and emergency appointment (which are depicted in shades of green, and represent the higher urgency to seek care categories, on the right-hand side of the *x* axis of the percentage of owners that reported on each clinical sign). Dog owners were divided into two groups, including the opinion of dog owners whose dog had not experienced the sign previously (owner not experienced), and owners’ dogs who had experienced the sign (owner experienced). The percentage figure on the left-hand side is the sum of the lower urgency to seek care categories, and the percentage figure on the right is the sum of the higher urgency to seek care categories.

**Figure 7:**
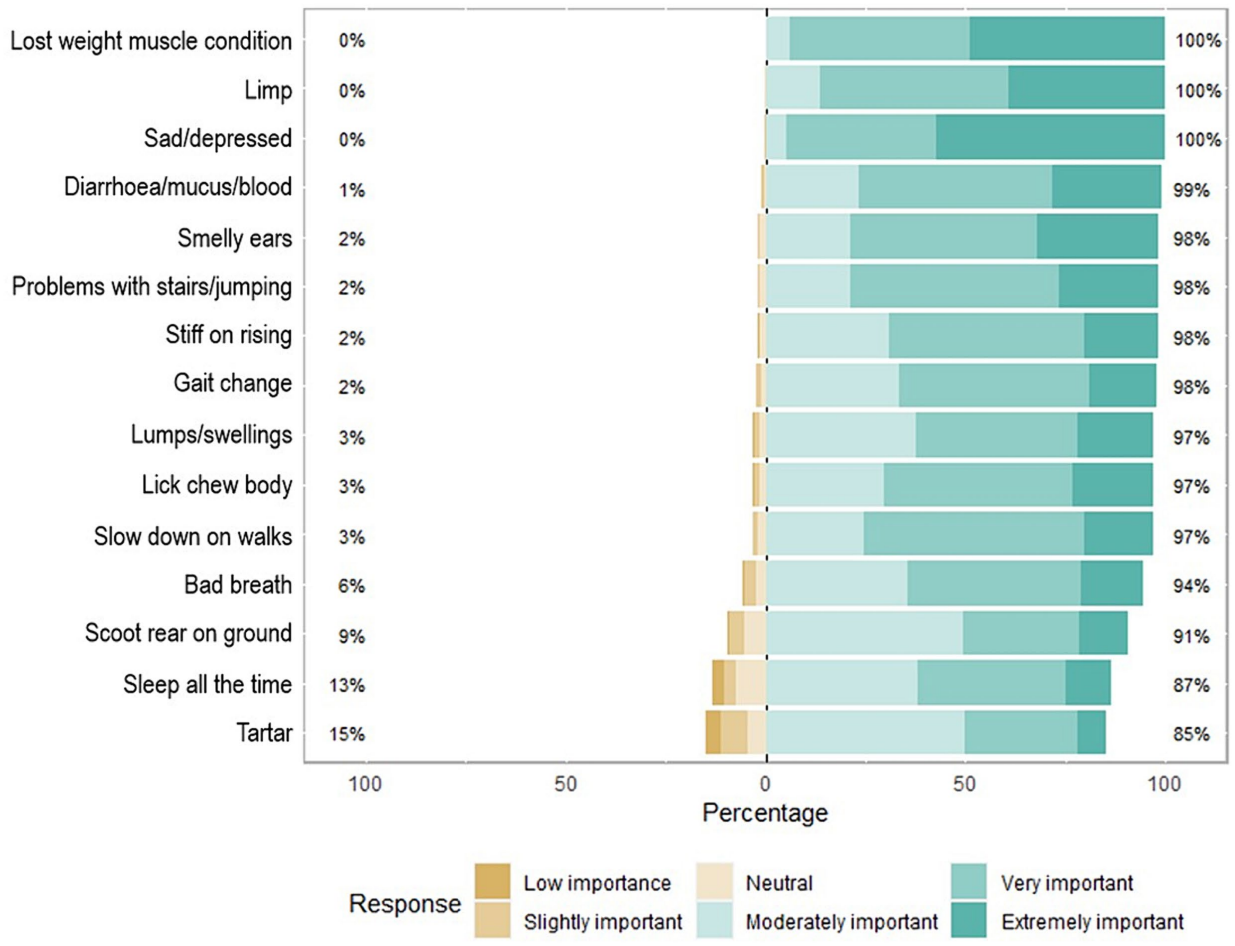
Diverging stacked bar chart for a six-point Likert scale question on how important veterinary professionals think it is for owners to seek veterinary advice for their senior dogs for the 15 most common clinical signs. The importance for owners to seek veterinary advice was categorised into a six-point Likert scale divided into lower and higher importance including: low importance, slightly important, and neutral (which are all depicted in shades of brown and represent the lower importance for owners to seek veterinary advice categories, on the left hand side of the *x* axis of percentage of veterinary professionals that reported on each clinical sign), and the categories moderately important, very important, and extremely important (which are depicted in shades of green, and represent the higher importance for owners to seek veterinary advice categories, on the right hand side of the x axis of percentage of veterinary professionals that reported on each clinical sign). The percentage figure on the left-hand side is the sum of the lower urgency to seek care categories, and the percentage figure on the right is the sum of the higher urgency to seek care categories.

Dog owners who stated that their dog had experienced a clinical sign typically reported less urgency to seek veterinary care than owners whose dog had never experienced it and responded to a hypothetical question of what they would do if they noticed this sign in their dog (Figure 6). The majority of veterinary professionals (85–100%) thought that it was moderately to extremely important for owners of senior dogs to seek veterinary advice for all 15 of the most common clinical signs (Figure 7). In contrast, there were particular clinical signs that fewer than 70% of owners would seek veterinary care for their dog within a week (Figure 6), including dental issues (bad breath and tartar), musculoskeletal issues (problem with stairs/jumping, slowing down on walks and stiff on rising), and sleeping for the majority of the time.

### 3.6 Dog owners attribute clinical signs to just old age

**Figure 8:**
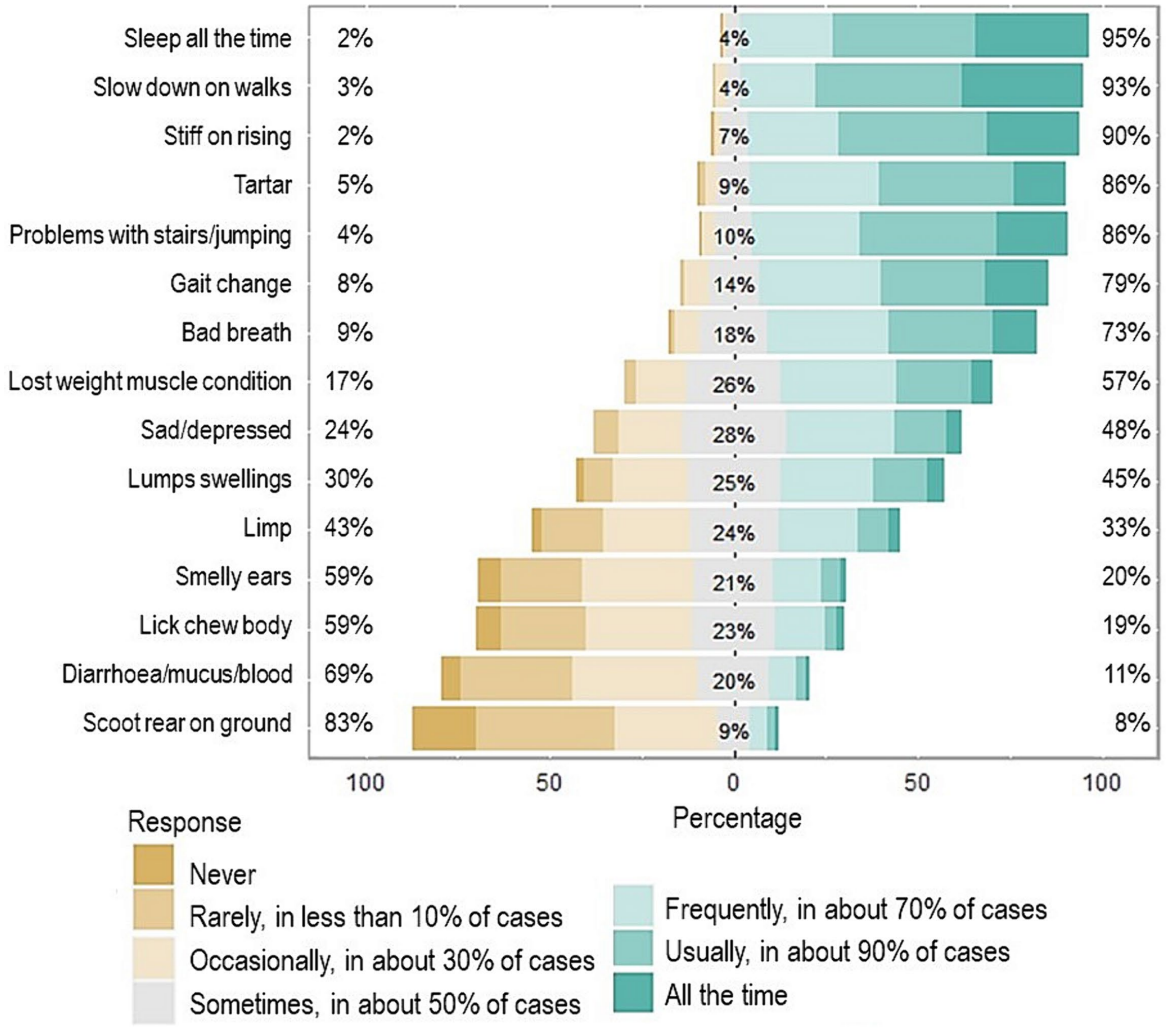
Diverging stacked bar chart for how often veterinary professionals believe owners attribute each of the 15 most common clinical signs to just old age. The frequency of how often veterinary professionals believed owners attributed signs to old age was categorised into a seven-point Likert scale divided into three groups low, neutral and high and included: Never, rarely, in less than 10 percent of cases, and occasionally, in about 30% of cases (which are all depicted in shades of brown and represent the lower frequency for how often veterinary professionals believe owners attribute the signs to old age, on the left hand side of the *x* axis of the percentage of veterinary professionals), and the category sometimes, in about 30% of cases (depicted in grey, representing the neutral frequency for how often veterinary professionals believe owners attribute the signs to old age, in the central part of the *x* axis), and the categories frequently, in about 70% of cases, usually, in about 90% of cases, and all the time (depicted in shades of green and represent the higher frequency for how often veterinary professionals believe owners attribute the signs to old age, in the right-hand side of the *x* axis of the percentage of veterinary professionals). The percentage figure on the left-hand side is the sum of the veterinary professionals who believed owners were less likely to categorise the sign as just old age, in the centre the percentage represents the neutral (or 50% of the time) category and the percentage figure on the right is the sum of the veterinary professionals who believed owners were more likely to categorise the sign as just old age.

**Figure 9:**
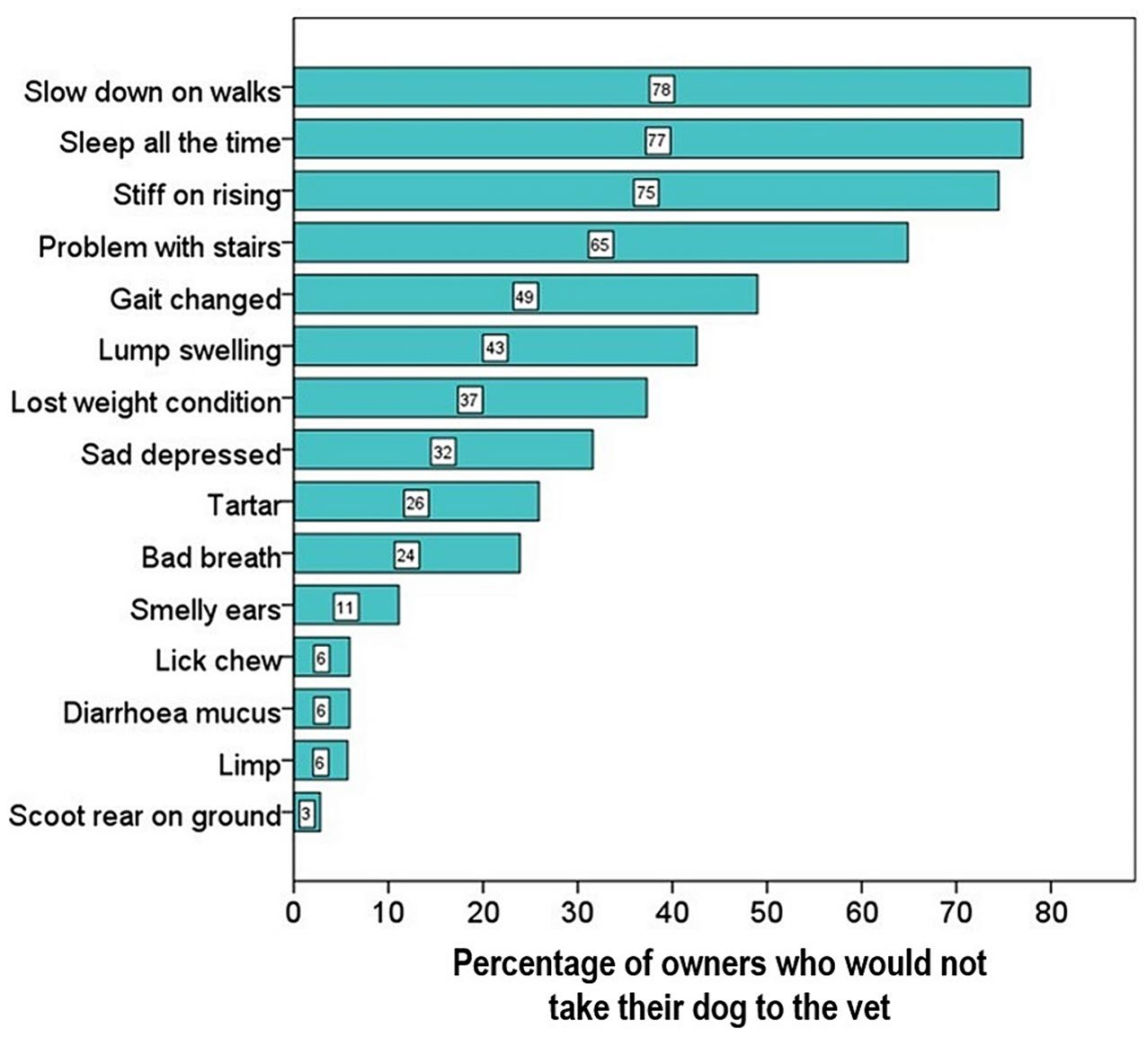
Stacked bar chart displaying the percentage of owners who would not take their dog to the vet as they believe the clinical sign was a normal part of the ageing process. The 15 most common clinical signs are shown in order of increasing importance. The numbers in the bars indicate the actual percentage of owners who responded that they would not take the dog to the vet, as they believe the sign is normal for older dogs.

The signs that veterinary professionals believed owners most-commonly associated with old age rather than illness included sleeping all the time, slowing down on walks, being stiff on rising, and the presence of calculus (tartar) (Figure 8). One of the main reasons for owners not taking their dogs to their veterinary practise was because they believed the clinical sign was a normal part of the ageing process (Figure 9) and owners’ opinions were broadly in line with veterinary professionals’ perceptions of them.

### 3.7 Future senior dog healthcare resource design

Five hundred and sixty owners (89%) and 249 veterinary professionals (82%) reported that they would be willing to fill in (or go through together with owners) a short questionnaire to help identify potential problems; however, veterinary surgeons were less willing than veterinary nurses [192 (78%) vs. 57 (95%)] (Table 4). Most veterinary professionals (164, 66%) preferred to briefly go through the submitted responses themselves; however, veterinary nurses (17, 30%) more often preferred the nursing team to be responsible for the questionnaire in comparison to veterinary surgeons (18, 9%). Thirty-three veterinary professionals (13%) would prefer questionnaire analysis to be automated and any potential problems flagged by the computer system and 16 (7%) already utilised a questionnaire in their consults. Of the veterinary professionals who did not think that owner questionnaires were a good idea (53, 17%), many agreed in principle but had reservations, including lack of time (36, 68%), previous bad experience with questionnaires and/or a preference for face to face (12, 23%), low owner and veterinary professional compliance (4, 8%), and lack of owner digital literacy (1, 2%).

**Table 4:** Results from the dog owner and veterinary professional questionnaires examining the design and implementation of a future senior dog healthcare resource.

Question	Categories	DO (*N*, %)	VP (*N*, %)	*VS* (*N*, %)	VN (*N*, %)	Chi-square, (*p*)
Would you be willing to fill in a short questionnaire before your next visit to the practise? (DO) Do you think it would be a good idea to ask owners of senior dogs to fill in a short health questionnaire before their visit? (VP)	No	69 (11)	53 (18)	50 (21)	3 (5)	8.2, (**0.004**) VS VN
	Yes	560 (89)	249 (82)	192 (79)	57 (95)	37.6, (**<0.001**) DO VS VN
	Missing	4	3	3	0	
If yes, how should the questionnaire be run?	Automated, any potential problems flagged		33 (13)	25 (13)	8 (14)	20.7, (**<0.001**) VS VN
	Nursing team responsible		35 (14)	18 (10)	17 (30)	
	Personally willing to briefly go through in consult		164 (66)	139 (72)	25 (45)	
	Already do this at our practise in some form		16 (7)	10 (5)	6 (11)	
How often do you think owners should fill in a questionnaire?	Every month	138 (25)	31 (13)	22 (12)	9 (18)	2.6, (0.462) VS VN 25.6, (**<0.001**) DO VP
	Quarterly	11 (2)	12 (5)	9 (5)	3 (6)	
	Every 6 months	232 (42)	130 (57)	107 (59)	23 (47)	
	Once a year or every 2 years	174 (31)	57 (25)	43 (24)	14 (29)	
	Missing	78	75	64	11	
How should owners fill in such a questionnaire? (Multiple options possible for VP, but not for DO)	Paper copy	25 (5)	29^*^ (8)	25^*^ (9)	4^*^ (5)	1.7, (0.630) VS VN 37.2, (**<0.001**) DO VP
	Email	50 (9)	80^*^ (22)	63^*^ (21)	17^*^ (22)	
	Online	291 (52)	152^*^ (41)	117^*^ (40)	35^*^ (46)	
	Mobile app	192 (34)	109^*^ (29)	89^*^ (30)	20^*^ (27)	
	Missing	75	69^*^	58^*^	11^*^	

DO, Dog owner; VP, Veterinary professional; VS, Veterinary surgeon; VN, Veterinary nurse. Chi-squared tests were carried out to determine whether veterinary surgeons and veterinary nurses differed in their opinions (the category missing was not included in the analyses). If veterinary surgeons and nurses did not differ, where possible dog owners were compared with veterinary professionals, and if they did differ, all three groups were compared (dog owners, veterinary surgeons, and nurses). ^*^For the question, ‘How should owners fill in such a questionnaire?’ Veterinary professionals could select multiple options, which explains why the total count exceeds the sample size.

Veterinary professionals and owners agreed on the most popular methods to complete the questionnaire, including the use of a secure internet form or a mobile application, however more veterinary professionals preferred emails, and owners an online form. Both most-commonly felt it should be completed every 6 months, however, some owners more frequently preferred every month and once a year than veterinary professionals (Table 4).

## 4 Discussion

In the current United Kingdom study, veterinary professionals felt that senior dogs require a different consultation approach to younger dogs, but most did not have a specific practise protocol. Nearly a third of all dogs were not vaccinated in the preceding year, with many being older than those that were vaccinated, and their owners believed they no longer needed vaccinations because they were old. In contrast, veterinary professionals considered booster vaccination appointments to be very important. Some owners believed that a senior dog should only visit the vet if they got sick, and 16% of owners of dogs of all ages had not had any contact with their veterinary practise in the previous year. Only 10% of dogs considered ‘old’ by their owners had attended a senior wellness clinic or examination, despite 14% of the surveyed veterinary professionals’ practises offering them. In summary, we found that older dogs were more likely to suffer from health issues and require medication than younger dogs, but they were less likely to receive preventive care or to be enrolled on a healthcare plan or be insured, highlighting the need for new interventions targeting ageing dogs. Most owners and veterinary professionals would be willing to fill in/go through a short questionnaire to help identify potential problems before/during a consultation. Filling in a secure online form or mobile app every 6 months was the preferred method, although there were some concerns about digital literacy.

The most common health conditions reported by the owners in dogs of all ages included orthopaedic (47%), dental (35%), integument (29%), sensory loss (hearing 29%, sight 27%), neoplasia (19%), digestive (18%), and neurological (15%). Many owners reported that sensory loss and dementia had not been officially diagnosed by their veterinary surgeon. The top five most common clinical signs reported by the owners were related to possible orthopaedic disorders at 57% (slowing down on walks, being stiff on rising, and problems with stairs/jumping), dental issues at 52% (tartar) and digestive at 44% (diarrhoea).

Other studies have found lower proportions of diagnoses and lower median age of death than our study, but this may be due to younger study populations (32, 33), higher proportions of pure-breds (34–37), or different countries cultural differences in dog treatment and care (32). Our findings are however similar to Salvin et al. (38) study that had a comparable sample median age (11.7 years).

Interestingly, we observed that 15% of dogs were suspected to be affected by canine dementia by their owners. Using a questionnaire, Salvin et al. (38) estimated the prevalence of Canine cognitive dysfunction (CCD) as 14.2%, but the rate of veterinary diagnosis was only 2%. The current study found a slightly higher diagnosis rate at 7%, which is encouraging, as this suggests that more veterinary professionals and owners are becoming aware of this problem. However, 50% of dogs that were affected did not receive a formal diagnosis, which confirms our previous study results that owners of senior and geriatric dogs reported dementia like behaviour in interviews, but did not inform their veterinary surgeon, or if they did some owners remarked that the veterinary surgeon did not always know how to treat it (15). Duxbury et al. (39) found that owners of senior dogs were more likely to identify behaviours of concern when prompted with a screening questionnaire, than they were to volunteer this information during a routine examination. Questionnaires also provide a means of tracking behavioural and physical signs over time, as changes in senior dogs can be detected over a 6-month period, and rates of deterioration may indicate the severity of decline, and whether interventions or further diagnostics are necessary (18, 40).

Our findings support that most owners believe that older dogs should only see a veterinary surgeon once a year in comparison to veterinary professionals’ recommendations of every 6 months. Many older dogs are not vaccinated, supporting other literature (41). Unless these dogs were receiving ongoing treatment for chronic conditions, they may not be seen by a veterinary surgeon for long periods. Whilst most owner’s trust their veterinary surgeon to provide the care needed (42), if the owner does not perceive the dog to have a problem, they may elect not to take them to a veterinary practise. Belshaw et al. (11) found that adult pet vaccination consultations rarely discuss preventive healthcare or owner education, which may help to explain why owners become disengaged with the traditional ‘annual’ vaccination and health check as their dog becomes older. For all senior and geriatric dogs, especially in cases when vaccines are not due or required, or owners are against vaccination in older dogs, veterinary professionals should be encouraged/advised to schedule a 6-monthly senior wellness check and set aside the time needed to go through all the most common issues including behavioural and physical. Vaccinations are arguably not the most important reason for the visit, and effective education and marketing is key to improving client compliance and therefore patient welfare (20).

Preventative education is important because knowing when to take their dog to the vet requires owners to be knowledgeable not only about their dogs’ normal behaviour and deviations from this, but also with what changes might be expected with age, and whether these changes indicate ‘normal’ ageing, or might suggest an underlying problem. Our findings support that owners are often unable to recognise early clinical signs of disease (13, 14, 43). Studies have shown that owners often need to be prompted to report signs, and the use of screening tools with a veterinary professional is a vital part of a preventative strategy (39, 44), as some signs may not be detectable without a veterinary exam and/or laboratory screening. Owners perceived urgency to seek veterinary care also appeared to be lower than the rated importance by veterinary professionals for most clinical signs, especially for orthopaedic, dental, sarcopenia, and excessive sleeping issues. The findings that owners who had previously experienced that clinical sign reported less urgency to seek veterinary advice than those who had not, indicates either a recall bias in the owner, as they may have forgotten how long they waited before seeking help, or, after initially taking a dog to the veterinary surgeon, owners may have been more confident in managing the clinical sign at home if it occurred again, without the need for veterinary intervention. The most common clinical signs often ignored by owners included halitosis (bad breath), dental calculus (tartar), slowing down on walks, problems climbing stairs or jumping, and sleeping all the time, which were also signs that owners commonly believed were a normal part of the ageing process or ‘just old age’. These signs can indicate underlying health issues such as the progression of dental disease and osteoarthritis (19, 45).

Our findings support previous observations that in traditional veterinary practises’ senior long-term health and patient well-being plans are uncommon (8). In the current study, only around one in 10 ran clinics, and 10% of owners of older dogs had attended one. However, many owners were not aware of the existence of senior wellness clinics, which may explain poor uptake, and encouragingly, 63% said they would attend now or in the future if the clinic was free. Since veterinary professionals indicated that time was a limiting factor on implementing senior wellness clinics, practise managers should explore the benefits of implementing and advertising a senior wellness programme to increase client engagement and the quality of life and longevity of older dogs. Veterinary nurses can run senior wellness clinics, which can pick up any concerns that can then be followed up in an appointment with a veterinary surgeon (20). Pre-booking and scheduling each patient for a senior wellness exam would allow for the allocation of the time required for evaluation and client counselling and would increase owner compliance. Many owners are willing to invest the time and commitment necessary to prevent and manage chronic issues, and prefer a more proactive wellness approach to the traditional reactive sick animal ‘fix it shop’ (8).

The current study provided important information exploring dog owner and veterinary professional expectations, experiences, and attitudes to ageing in dogs, but has some limitations. Although owners of any aged dog could take part, participants were informed that the research was specifically focused on senior dog care and so they may have been particularly interested and invested in this topic and therefore represent an especially conscientious sample. Male participants were particularly difficult to recruit, as is found in many questionnaires and qualitative studies involving animals (46). The questionnaires were quite long and complex, and many participants did not finish, and/or did not answer some questions. Some of the questions required owners to remember events, which could have been quite some time ago in the life of their dog, and so recall bias could be a significant issue. In some cases, it was difficult to directly compare the owner and veterinary professional responses, as due to error they were not asked identically (for example owners were asked when their dog became ‘old’ and veterinary professionals when a dog becomes ‘senior’). Regarding the generalisability (external validity) of the study, many of the results of our earlier studies (15, 23) were confirmed, however, for some groups, for example veterinary nurses, small sample sizes in the current study require future studies to examine the applicability of our results.

Online questionnaires have the advantage of a wide geographical scope of the whole of the United Kingdom, but also the disadvantages of possibly lacking more diverse participant groups (e.g., groups from nonprofessional backgrounds and people with low levels of education and/or computer literacy) (47). Due to the large number of topics covered and the exploratory nature of the study, descriptive and univariate analyses were utilised, which cannot control for possible confounding variables. This was a hypothesis-generating study and therefore a larger confirmatory study is needed in the future. Due to the correlational nature of the study design, it was not possible to determine the cause and effect of the various associations found.

## 5 Conclusion

In the current study, owners and veterinary professionals differed in their opinions about the need for veterinary care, suggesting new educational initiatives, and more effective communication is required. Owners regularly attributed clinical signs in senior and geriatric dogs to normal ageing, and thus may not mention them to their veterinary professional. Findings from this study have been used to support the design of a new resource, which can facilitate communication between owners and veterinary professionals. A checklist of common missed clinical signs for use pre-veterinary appointment would be supported by both owners and veterinary professionals surveyed in the current study. The BSAVA PetSavers Ageing Canine Toolkit (ACT) leaflet and poster (48) are currently in use in first opinion practise, and feedback is being collected to measure the toolkits impact on owners, veterinary professionals, and senior dogs. Screening tools and toolkits have the potential to increase owner understanding and compliance, and through repeated application over time and implementation of necessary interventions, improve patient welfare and health span.

## Data availability statement

The raw data supporting the conclusions of this article will be made available by the authors, without undue reservation.

## Ethics statement

The studies involving human participants were reviewed and approved by the University of Liverpool Veterinary Ethics Committee (ethical approval project code VREC1122, for the veterinary professional survey; ethical approval project code VREC1109, for the dog owner survey). The participants provided their written informed consent to participate in this study.

## Author contributions

LW: Conceptualization, Data curation, Formal Analysis, Funding acquisition, Investigation, Methodology, Project administration, Resources, Supervision, Validation, Visualization, Writing – original draft, Writing – review & editing. AR: Conceptualization, Funding acquisition, Methodology, Writing – review & editing. ZB: Conceptualization, Funding acquisition, Methodology, Writing – review & editing. JJ: Writing – review & editing. EK: Conceptualization, Funding acquisition, Methodology, Validation, Writing – review & editing. AG: Funding acquisition, Methodology, Validation, Writing – review & editing. CW: Conceptualization, Formal Analysis, Funding acquisition, Methodology, Resources, Supervision, Validation, Visualization, Writing – review & editing, Investigation.
