# Supplementary material for: Cross-sectional United Kingdom surveys demonstrate that owners and veterinary professionals differ in their perceptions of preventive and treatment healthcare needs in ageing dogs
Source: Front Vet Sci. 2024 Apr 4;11:1358480. doi: 10.3389/fvets.2024.1358480 (PMC11024473; doi:10.3389/fvets.2024.1358480)
Supplement: Supplementary file 1 [file Table_1.DOCX]

Supplementary Material A

# Demographics

Supplementary Table 1. Dog and owner categorical demographics, N and percentage of full sample.

| **Title/description** | **Categorical variable labels:** | **N** | **Percentage** |
| --- | --- | --- | --- |
| Number of dogs living in household | None | 31 | 4.90 |
|  | One | 300 | 47.39 |
|  | Two | 141 | 22.27 |
|  | Three | 54 | 8.53 |
|  | Four or more | 45 | 7.11 |
|  | Prefer not to say | 62 | 9.79 |
| Dog passed away | No | 461 | 72.83 |
|  | Yes | 162 | 25.59 |
|  | Prefer not to say | 10 | 1.58 |
| Dog described as old | No | 236 | 37.28 |
|  | Yes | 384 | 60.66 |
|  | Prefer not to say | 13 | 2.05 |
| Medical insurance | Yes | 336 | 53.08 |
|  | No | 297 | 46.92 |
| Currently on medication | Yes | 310 | 48.97 |
|  | No | 318 | 50.24 |
| Size of dog | Large/Giant | 189 | 29.86 |
|  | Medium | 284 | 44.87 |
|  | Toy/Small | 160 | 25.28 |
| Breed | Crossbreed | 114 | 18.01 |
|  | Purebred | 370 | 58.45 |
|  | Mixed breed | 101 | 15.96 |
|  | Prefer not to say | 48 | 7.58 |
| Sex | Female | 319 | 50.39 |
|  | Male | 304 | 48.03 |
|  | Prefer not to say | 10 | 1.58 |
| Neuter status | Intact | 101 | 15.96 |
|  | Neutered | 531 | 83.89 |
|  | Prefer not to say | 1 | 0.16 |
| Owner sex | Female | 584 | 92.26 |
|  | Male | 47 | 7.42 |
|  | Prefer not to say | 2 | 0.32 |
| Owner age | 18 - 29 | 92 | 14.53 |
|  | 30 - 39 | 142 | 22.43 |
|  | 40 - 49 | 151 | 23.85 |
|  | 50 - 59 | 139 | 21.96 |
|  | >60 | 109 | 17.22 |
| Owner education | GCSE | 72 | 11.37 |
|  | A Level | 79 | 12.48 |
|  | Nursing | 43 | 6.79 |
|  | Diploma | 86 | 13.59 |
|  | Degree | 183 | 28.91 |
|  | Higher Degree | 158 | 24.96 |
|  | Prefer not to say | 12 | 1.90 |
| Owner income | <£20,000 | 109 | 17.22 |
|  | £20,001 - £30,000 | 114 | 18.01 |
|  | £30,001 - £40,000 | 88 | 13.90 |
|  | £40,001 - £60,000 | 118 | 18.64 |
|  | > £60,000 | 118 | 18.64 |
|  | Prefer not to say | 86 | 13.59 |

**Supplementary Table 2.** Veterinary professional categorical demographics, N and percentage of full sample.

| **Title and description** | **Categorical variable labels:** | **N** | **Percentage** |
| --- | --- | --- | --- |
| Profession | Veterinary surgeon | 245 | 80.33 |
|  | Veterinary nurse | 60 | 19.67 |
| Sex | Female | 255 | 83.61 |
|  | Male | 45 | 14.75 |
|  | Prefer not to say | 5 | 1.64 |
| Age | Less than 30 | 85 | 27.87 |
|  | 30 - 40 | 81 | 26.56 |
|  | 41 - 50 | 65 | 21.31 |
|  | 51 - 60 | 52 | 17.05 |
|  | >60 | 21 | 6.89 |
|  | Prefer not to say | 1 | 0.33 |
| Ethnicity | White | 288 | 94.43 |
|  | Mixed | 8 | 2.62 |
|  | Asian | 3 | 0.98 |
|  | Black | 1 | 0.33 |
|  | Prefer not to say | 5 | 1.64 |
| Length of service | Under 1 year | 14 | 4.59 |
|  | 1 - 5 years | 75 | 24.59 |
|  | 6 - 10 years | 47 | 15.41 |
|  | 11 - 20 years | 59 | 19.34 |
|  | > 20 years | 110 | 36.07 |
| Practice category (can select more than one option) | Corporate | 158 | 48.17 |
|  | Independent | 134 | 40.85 |
|  | Charity | 7 | 2.13 |
|  | Referral | 16 | 4.88 |
|  | Vet school | 4 | 1.22 |
|  | Other | 9 | 2.74 |
| Employment (can select more than one option) | Self-employed | 39 | 11.89 |
|  | Locum | 41 | 12.50 |
|  | Work at more than one practice | 44 | 13.41 |
|  | Employed at one practice | 204 | 62.20 |
| Practice type | Mixed/large | 37 | 12.13 |
|  | Small animal | 268 | 87.87 |

# Perceptions about senior dog healthcare and wellness checks

**Supplementary Table 3.** Results from the dog owner questionnaire examining practice protocols, health plans, senior wellness clinics and own beliefs concerning the healthcare of senior dogs.

|  | Categories | N | Percentage |
| --- | --- | --- | --- |
| Did you consult with a veterinary practice/hospital/ online vet in the last 12 months (in person or via remote consultation)? | No | 104 | 16.43 |
|  | Yes | 519 | 81.99 |
|  | Missing/don’t know | 10 | 1.58 |
| How many times did your dog go physically to the vet in the last 12 months (or the 12 months before your dog passed away) | Once | 86 | 16.57 |
|  | Twice | 117 | 22.54 |
|  | Three – five times | 171 | 32.95 |
|  | 6 – 10 times | 78 | 15.03 |
|  | > 10 times | 43 | 8.29 |
|  | Remote consult | 9 | 1.73 |
|  | Missing | 15 | 2.89 |
| Was the consultation/s for –  (can select more than one option) | Routine health appointment (vaccination, anal gland check, nail clip, medication recheck) | 352 | 44.22 |
|  | New health condition/illness | 288 | 36.18 |
|  | Advice on euthanasia/end of life care | 87 | 10.93 |
|  | Other | 69 | 8.67 |
| Do/did you belong to a health plan at your veterinary practice or online | No | 470 | 74.25 |
|  | Yes | 161 | 25.43 |
|  | Missing | 2 | 0.32 |
| Have you ever attended a senior dog wellness clinic/exam at your veterinary practice (with vet/ veterinary nurse) for this dog? | Dog not old | 168 | 26.54 |
|  | No | 303 | 47.87 |
|  | No, but yearly general exam performed | 113 | 17.85 |
| Have you ever attended a senior dog wellness clinic/exam at your veterinary practice (with vet/ veterinary nurse) for this dog?  Are senior wellness clinics offered at your practice?  Would you be interested in attending one? | Yes | 46 | 7.27 |
|  | Missing | 3 | 0.47 |
|  | No | 80 | 28.57 |
|  | Don’t know | 200 | 71.43 |
|  | No | 129 | 21.98 |
| Are senior wellness clinics offered at your practice? | Yes, but only if it was free | 115 | 19.59 |
|  | Yes | 251 | 42.76 |
| Would you be interested in attending one?  Was this dog vaccinated in the last year?  Why was your dog not vaccinated? | Missing | 92 | 15.67 |
|  | No | 175 | 27.65 |
|  | Yes | 458 | 72.35 |
|  | My dog has never been vaccinated | 7 | 4.00 |
| Was this dog vaccinated in the last year? | My dog received puppy vaccinations only | 50 | 28.86 |
|  | Vaccinated every 3 years | 2 | 1.14 |
| Why was your dog not vaccinated? | Due to medical condition/illness/anxiety | 15 | 8.57 |
|  | Titre test, and only vaccinate if needed | 29 | 16.57 |
|  | Older dogs don’t need vaccinations | 57 | 32.57 |
|  | Covid and/or finances | 13 | 7.43 |
|  | Missing | 2 | 1.14 |
|  |  |  |  |
|  |  |  |  |
|  |  |  |  |
