# Supplementary material for: Cross-sectional United Kingdom surveys demonstrate that owners and veterinary professionals differ in their perceptions of preventive and treatment healthcare needs in ageing dogs
Source: Front Vet Sci. 2024 Apr 4;11:1358480. doi: 10.3389/fvets.2024.1358480 (PMC11024473; doi:10.3389/fvets.2024.1358480)
Supplement: Supplementary file 2 [file Table_2.DOCX]

Supplementary Material B

# PetSavers Old Age Pets Owner Survey

Block: Introduction (1 Question)

Standard: Screening questions (2 Questions)

Branch: New Branch

If Do you own one or more dogs, or have you done so in the last year (for example you owned a dog wh... No Is Selected

EndSurvey:

Branch: New Branch

If Are you an adult (18 years or over) who lives in the United Kingdom? No Is Selected

EndSurvey:

Standard: Dog status (2 Questions)

Standard: Dog Demographics (44 Questions)

Block: Deceased dog questions (6 Questions)

Standard: Dog clinical signs 1 (36 Questions)

Standard: Dog clinical signs 2 (15 Questions)

Standard: Dog clinical signs 3 (30 Questions)

Standard: Dog clinical signs 4 (25 Questions)

Standard: Dog clinical signs 5 (40 Questions)

Standard: Dog clinical signs 6 (20 Questions)

Standard: Dog clinical signs 7 (40 Questions)

Standard: Dog clinical sign 8 (35 Questions)

Standard: Caregiver burden (1 Question)

Standard: Owner demographic (9 Questions)

| Page Break |  |
| --- | --- |

Start of Block: Introduction and consent

Q1.1 You are being invited to participate in a research study. Before you decide whether to participate, it is important for you to understand why the research is being done and what it will involve. Please take time to read the following information carefully and feel free to ask us if you would like more information or if there is anything that you do not understand.

**1.                What is the purpose of the study?** The purpose of this study is to better understand how owners of dogs of all ages approach the healthcare of their dog, and what is their experiences of, and attitudes to, ageing in dogs. We are looking to recruit UK adults who currently own a dog or had a dog in the past year (for example your dog recently died). The findings will be used to develop a proforma and guidance tool for use in veterinary consultations with owners of senior dogs.

**2.                Who is involved in running this study?** PetSavers is the funder for this project and Dr Carri Westgarth is the principal investigator. Carri is a Senior Lecturer in Human-Animal Interaction at the University of Liverpool. Dr Lisa Wallis is the principal researcher on the project.

**3.                Why should I take part?** We would like dog owners from a wide range of different backgrounds, that own different dog types/breeds of different ages, including healthy dogs as well as dogs suffering from sensory loss and/or age-related diseases, to complete the survey. We are also asking owners who recently lost an older dog to participate as we are also interested in your experiences of caring for dogs at the end-of-life stage and through euthanasia or natural death. We understand that talking about these topics can be distressing; at the end of the survey, we provide links for relevant support groups for those who are affected.

**4.                Do I have to take part?** No. Participation is entirely voluntary, and participants are free to withdraw at any time during the questionnaire.

**5.                What will happen if I take part?** The survey will take you approximately 25 minutes to complete. After the first two questions, you are free to omit any of the questions if you wish. You will be asked some general information about your dog (demographics); about some common physical and behavioural signs that your dog might show; and some general information about yourself (demographics).

Your participation is voluntary. You may choose not to participate, and you may withdraw up until the survey is completed. If you decide not to participate or to withdraw from the study, you will not be penalised in any way.
Your participation remains anonymous. Minimal demographic information will be collected to protect your identity. No personal data will be collected in the main survey, and all data will be collected and stored safely in compliance with General Data Protection Regulation (GDPR) legislation.

**Contact information**: For further queries, please contact Dr Lisa Wallis, Epidemiology and Population Health and School of Veterinary Science, University of Liverpool, Leahurst, Chester High Road, Neston, Cheshire, CH64 7TE, Tel: 0151 795 1426, Email: lisa.wallis@liverpool.ac.uk

**Consent statement:**

- You understand the purpose of this study, and that you are able to ask questions about it at any time.
- You understand that you are free to withdraw your consent for involvement up until reaching the end of the survey. You understand that if you decide to withdraw from the study, you will not be penalised in any way.
- You understand that your name and identifying information will NOT appear in any published document relating to this study, as all information collected in the main survey is completely anonymous, and minimal demographic information is collected to protect your identity.
- You understand that the data collected will - though fully anonymised - appear in publications and reports relevant to the purpose of this study and may be used for future ethically approved research.
- You understand that your responses will be confidential and remain anonymous.
- You understand that no personal data will be collected, and all data will be collected and stored safely in compliance with GDPR legislation.
- You understand that you may ask for the results of the study on its completion using the above contact information.

**Please click 'Yes' if you have read and understood the above and give your informed consent to participate in this study.**

- Yes

End of Block: Introduction and consent

Start of Block: Screening questions

Q2.1 Are you an adult (18 years or over) who lives in the United Kingdom?

- Yes (1)
- No (2)

Q2.2 Do you own one or more dogs, or have you done so in the last year (for example you owned a dog who died recently)?

- Yes (1)
- No (2)

End of Block: Screening questions

Start of Block: Dog status

Q3.1 How many dogs are currently living in your household?

▼ None (1) ... Five or more (6)

Q3.2 If your oldest dog passed away in the past year, would you be willing to complete the survey for that dog, thinking about the last three months of your dog's life?

- Yes – my dog died in the past year and I would be willing to complete the survey for this dog. (1)
- Yes - my dog died in the past year, but I would rather complete the survey for a different dog. (2)
- No – my dog/s are still alive. (3)

End of Block: Dog status

Start of Block: Dog Demographics

Q4.1 In this section, we will ask you questions about your dog and your views on senior dog healthcare. If you have more than one dog, **please complete the survey for the oldest dog that you own**. If your oldest dog died in the past year, please complete the questions for that dog, thinking about the last three months of your dog's life, if you are happy to do so.

Q4.2 Which size is/was your dog?

- Toy (e.g. Chihuahua) (1)
- Small (e.g. Terrier) (2)
- Medium (e.g. Collie/Spaniel) (3)
- Large (e.g. Labrador/ German Shepherd Dog) (4)
- Giant (e.g. Great Dane) (5)

Q4.3 Please select the most appropriate statement about your dog:

- My dog is/was a pure breed (1)
- My dog is/was a cross breed of known parents (2)
- My dog is/was an unknown crossbreed (3)

Display This Question:

If Please select the most appropriate statement about your dog: = My dog is/was a pure breed

Q4.4 Which pure breed is/was your dog?

▼ Affenpinscher (1) ... Yorkshire Terrier (506)

Display This Question:

If Please select the most appropriate statement about your dog: = My dog is/was a cross breed of known parents

Q4.5 Please list the breeds in your crossbreed dog (the first four that you are sure about)

Breed 1

▼ Affenpinscher (1) ... Yorkshire Terrier (506)

Breed 2

▼ Affenpinscher (1) ... Yorkshire Terrier (506)

Breed 3

▼ No other breeds (1) ... Yorkshire Terrier (506)

Breed 4

▼ No other breeds (1) ... Yorkshire Terrier (506)

Q4.6 Is/was your dog male or female?

- Male (1)
- Female (2)

Q4.7 Is/was your dog neutered (spayed/castrated)?

- Yes (1)
- No (2)
- Don't know (3)

Q4.8 How old is this dog? (If your dog has recently died please select the age this occurred)

▼ Under 1 (1) ... Unknown (26)

| Page Break |  |
| --- | --- |

Q4.9 If you have more than one dog, **please complete the following information for the oldest dog that you own**. If your oldest dog died in the past year, please complete the questions for that dog, thinking about the last three months of your dog's life, if you are happy to do so.

Q4.10 What does this dog weigh in kilograms? (Or if your dog has passed, what did they weight in the three months before this occurred?)

▼ 1 (1) ... Don't know (66)

Display This Question:

If Which size is/was your dog? = Toy (e.g. Chihuahua)

Q4.11 Please rate your dog’s body shape from 1 to 9. Please use the provided picture guideline below to determine the body shape of your dog. If you are not sure in between two scores, please choose the higher score. (Tip: If your dog has long hair, then please feel under the hair to help determine body shape.) If your dog has passed away, please estimate their score in the three months before they passed.

▼ 1 (1) ... 9 (9)

Display This Question:

If Which size is/was your dog? = Toy (e.g. Chihuahua)

Q4.12
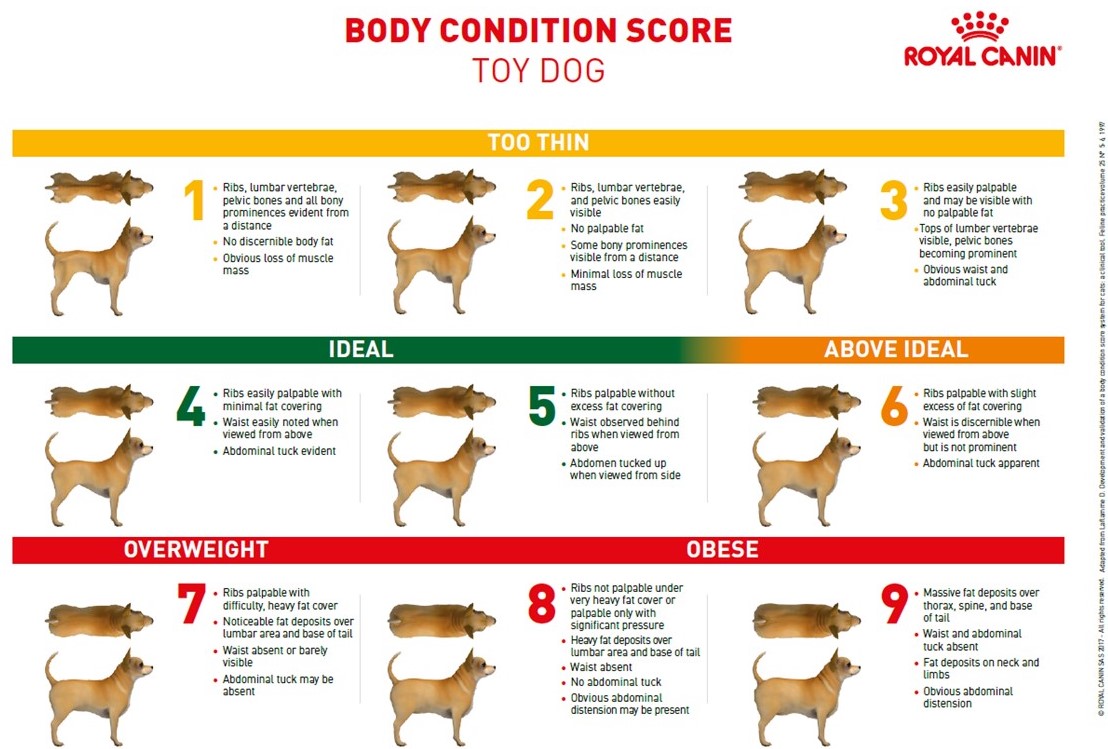


Display This Question:

If Which size is/was your dog? = Small (e.g. Terrier)

Q4.13 Please rate your dog’s body shape from 1 to 9. Please use the provided picture guideline above to determine the body shape of your dog. If you are not sure in between two scores, please choose the higher score. (Tip: If your dog has long hair, then please feel under the hair to help determine body shape.) If your dog has passed away, please estimate their score in the three months before they passed.

▼ 1 (1) ... 9 (9)

Display This Question:

If Which size is/was your dog? = Small (e.g. Terrier)

Q4.14
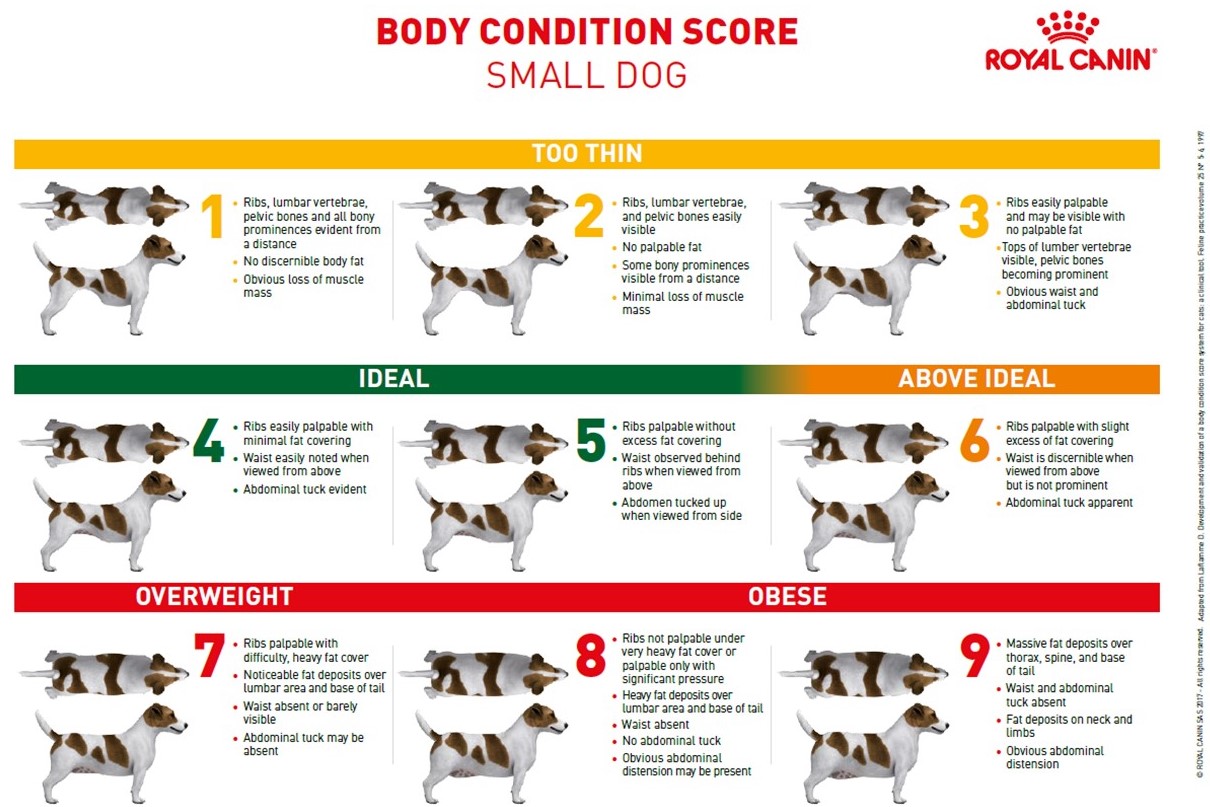


Display This Question:

If Which size is/was your dog? = Medium (e.g. Collie/Spaniel)

Q4.15 Please rate your dog’s body shape from 1 to 9. Please use the provided picture guideline above to determine the body shape of your dog. If you are not sure in between two scores, please choose the higher score. (Tip: If your dog has long hair, then please feel under the hair to help determine body shape.) If your dog has passed away, please estimate their score in the three months before they passed.

▼ 1 (1) ... 9 (9)

Display This Question:

If Which size is/was your dog? = Medium (e.g. Collie/Spaniel)

Q4.16
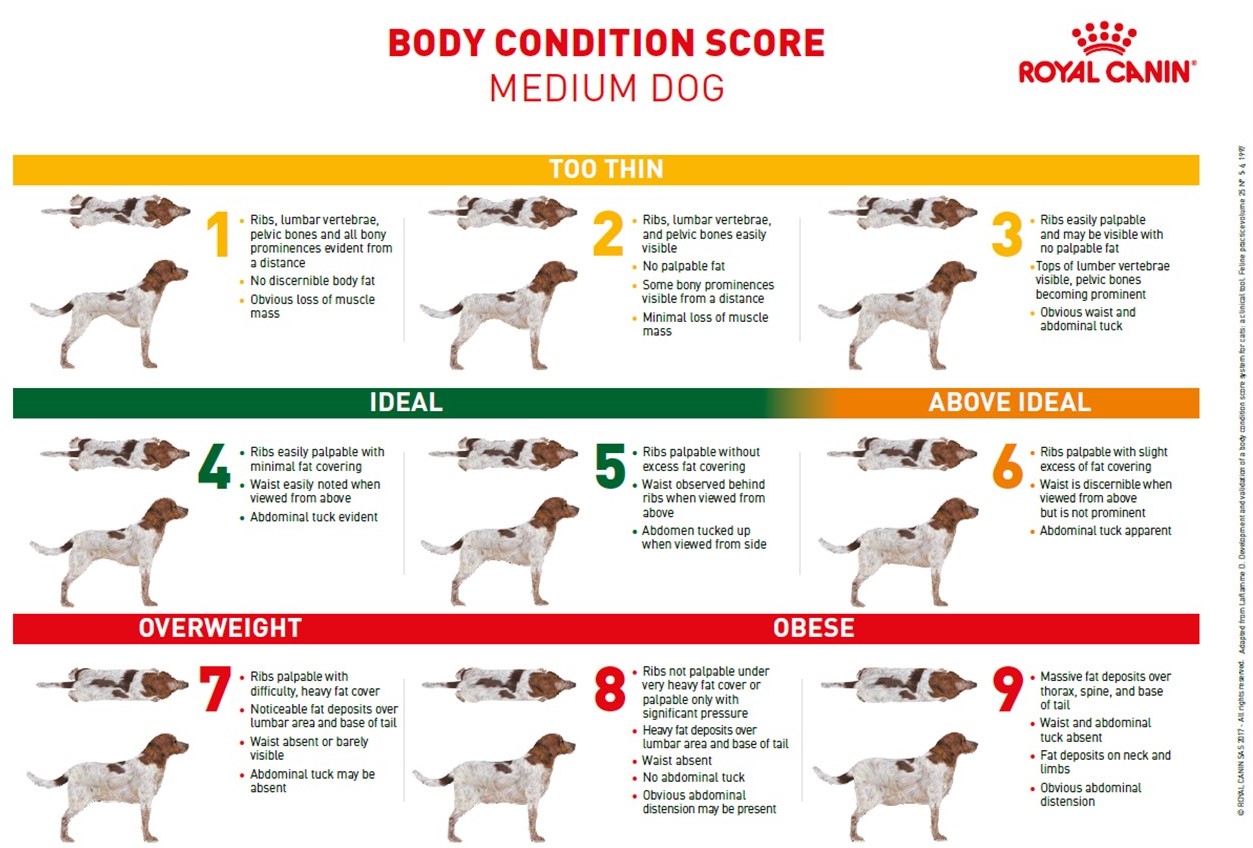


Display This Question:

If Which size is/was your dog? = Large (e.g. Labrador/ German Shepherd Dog)

Q4.17 Please rate your dog’s body shape from 1 to 9. Please use the provided picture guideline above to determine the body shape of your dog. If you are not sure in between two scores, please choose the higher score. (Tip: If your dog has long hair, then please feel under the hair to help determine body shape.) If your dog has passed away, please estimate their score in the three months before they passed.

▼ 1 (1) ... 9 (9)

Display This Question:

If Which size is/was your dog? = Large (e.g. Labrador/ German Shepherd Dog)

Q4.18
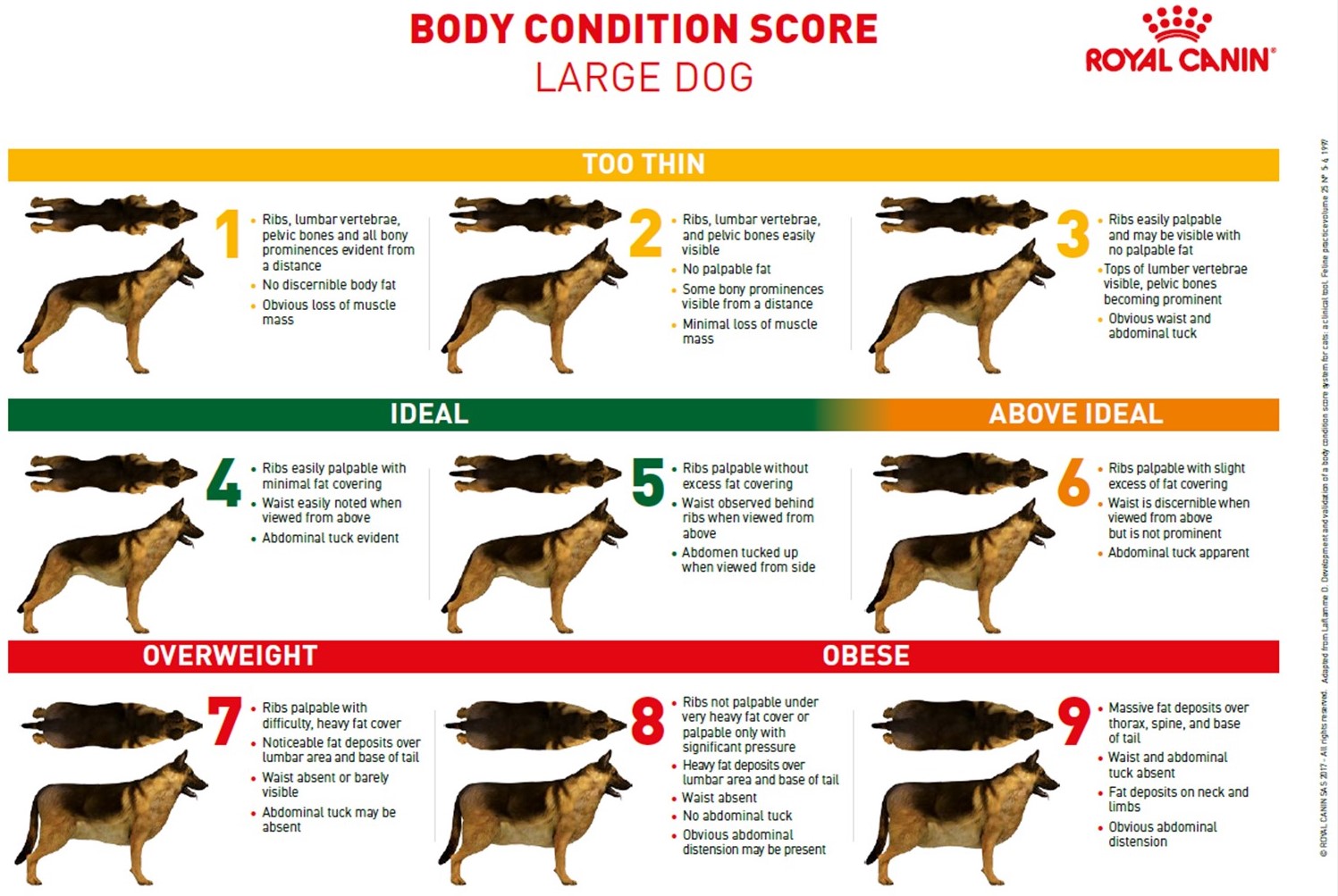


Display This Question:

If Which size is/was your dog? = Giant (e.g. Great Dane)

Q4.19 Please rate your dog’s body shape from 1 to 9. Please use the provided picture guideline above to determine the body shape of your dog. If you are not sure in between two scores, please choose the higher score. (Tip: If your dog has long hair, then please feel under the hair to help determine body shape.) If your dog has passed away, please estimate their score in the three months before they passed.

▼ 1 (1) ... 9 (9)

Display This Question:

If Which size is/was your dog? = Giant (e.g. Great Dane)

Q4.20
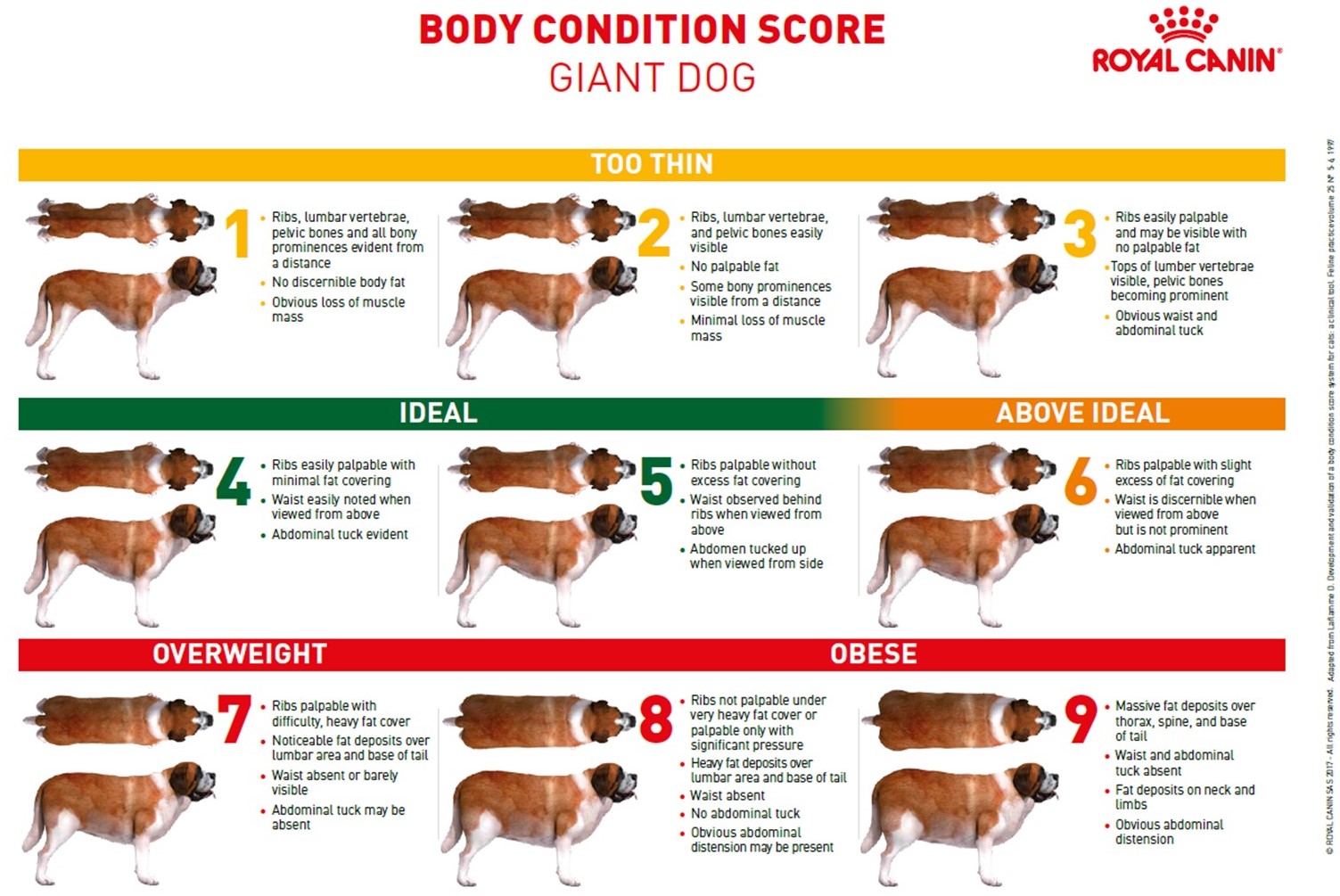


Q4.21 Do/Did you feed your dog a food that is specially formulated for senior dogs? If yes, please specify the brand and dog food name.

- Yes (1) ________________________________________________
- No (2)

|  |  |
| --- | --- |

Q4.22 If you have more than one dog, **please complete the following information for the oldest dog that you own**. If your oldest dog died in the past year, please complete the questions for that dog, thinking about the last three months of your dog's life, if you are happy to do so.

Q4.23 Rate your dog’s current health status, or if your dog has passed away, please answer thinking about the last three months of your dog’s life.

|  | 0 | 10 | 20 | 30 | 40 | 50 | 60 | 70 | 80 | 90 | 100 |
| --- | --- | --- | --- | --- | --- | --- | --- | --- | --- | --- | --- |

| 0 being in the worst possible health, and 100 – being in perfect health () | 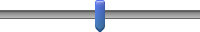 |
| --- | --- |

Q4.24
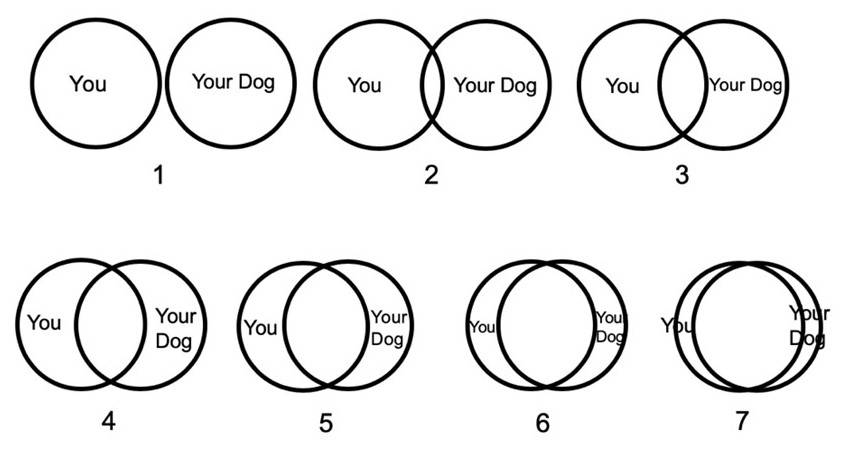


Q4.25 Looking at the picture above, select the pair of circles that best describes your relationship with your dog. If your dog has passed away, please think about your relationship in the three months before this occurred.

▼ 1 (1) ... 7 (7)

Q4.26 Was this dog vaccinated in the last year? (For example, first vaccinations or boosters for distemper, parvovirus and canine hepatitis every three years, or boosters for leptospirosis and kennel cough every year). If your dog has passed away, were they vaccinated in the year before this occurred?

- Yes, I vaccinate every year (1)
- Yes, I started vaccinating this year (2)
- Yes, this year they received a vaccine, but I don’t vaccinate routinely (3)
- No (4)
- I don’t know (5)

Display This Question:

If Was this dog vaccinated in the last year? (For example first vaccinations or boosters for distemp... = No

Q4.27 Why was your dog not vaccinated in the past year?

- My dog has never been vaccinated (1)
- My dog received puppy vaccinations only (2)
- I don’t think older dogs need vaccines as often/at all (3)
- I titer test, and only vaccinate if needed (4)
- Other (Please describe) (5) ________________________________________________

Q4.28 At what age do you think this dog became ‘old’?

▼ It hasn't yet become old (1) ... 23 (25)

Q4.29 If you have more than one dog, **please complete the following information for the oldest dog that you own**.  If your oldest dog died in the past year, please complete the questions for that dog, thinking about the last three months of your dog's life, if you are happy to do so.

Q4.30 Please go through the list of conditions below and indicate any that your dog has suffered over their lifespan. Select the column that best describes how your dog was diagnosed (by a vet, someone other than a vet (e.g., physiotherapist), or your dog has not been formally diagnosed, but you feel that your dog may have this condition, or your dog has never had this problem (or you don't know if they did (as they are/were a rescue)).

|  | Diagnosed by a vet (1) | Diagnosed by someone other than a vet (2) | Not formally diagnosed, but I feel my dog has it. (3) | My dog has never had this problem (4) |
| --- | --- | --- | --- | --- |
| Skin problems (skin sores, dry skin, rashes, redness, dandruff, hair loss) (2) |  |  |  |  |
| Kidney/liver disease (3) |  |  |  |  |
| Heart disease (degenerative mitral valve disease (DMVD), dilated cardiomyopathy (DCM), heart murmur, arrhythmias) (4) |  |  |  |  |
| Thyroid problems (under/over active) (6) |  |  |  |  |
| Epilepsy (fits/seizures) (7) |  |  |  |  |
| Cancer (Mast cell tumor, melanoma, lymphoma, bone cancer, hemangiosarcoma) (8) |  |  |  |  |
| Diabetes (9) |  |  |  |  |
| Gut problems (Inflammatory bowel disease/pancreatitis, colitis) (10) |  |  |  |  |
| Cushing’s disease, Addison’s disease (11) |  |  |  |  |
| Glaucoma (13) |  |  |  |  |
| Loss of eyesight/blind (cataracts (center of lens cloudy or gray), eye removed) (14) |  |  |  |  |
| Loss of hearing/deaf (15) |  |  |  |  |
| Orthopedic problems (fracture/break, Cranial Cruciate Ligament Rupture, hip/elbow dysplasia, osteochondrosis, Arthritis/osteoarthritis) (16) |  |  |  |  |
| Spinal problem (Intervertebral Disc Disease (IDD), Spondylosis) (19) |  |  |  |  |
| Breathing problems (Brachycephalic Obstructive Airway Syndrome (BOAS)) (23) |  |  |  |  |
| Dental disease (including where teeth have been cleaned or removed) (24) |  |  |  |  |
| Doggy dementia (canine cognitive dysfunction) (25) |  |  |  |  |
| Over-weight/Obese (27) |  |  |  |  |
| Vestibular disease/stroke (28) |  |  |  |  |
| Other (29) |  |  |  |  |

Q4.31 Is this dog currently taking medication prescribed by a vet? Or was it taking medication in the last three months before they passed? If yes - please write what medication your dog is/was taking.

- Yes (1) ________________________________________________
- No (2)

Q4.32 In addition to the medications prescribed by the veterinarian, did you try any other treatments in relation to the diseases diagnosed? These could also be recommended by veterinary professional or instigated by yourself as a general management tool to improve your dog’s health. Please select all that apply.

|  | Recommended by veterinary professional (1) | Owner instigated treatment (general management to improve health) (2) |
| --- | --- | --- |
| Weight management/control (1) |  |  |
| Home environment/lifestyle adaptations (rugs, ramps) (2) |  |  |
| Modified exercise routine (3) |  |  |
| Nutrition (changes in the main diet) (4) |  |  |
| Dietary supplements/vitamins/nutraceuticals) (5) |  |  |
| Homeopathic treatments (6) |  |  |
| Herbal therapy (7) |  |  |
| Acupuncture (8) |  |  |
| Tooth brushing (9) |  |  |
| Laser therapy (10) |  |  |
| Hydrotherapy (11) |  |  |
| Physiotherapy (12) |  |  |
| Other (13) |  |  |
| No other treatment/ therapy (14) |  |  |

Q4.33 Did you consult with a veterinary practice/hospital/online vet in the last 12 months (in person or via remote consultation (telephone/online chat))? (Or in the year before your dog passed away).

- Yes (1)
- No (2)
- I don't know/can't remember (3)
- Other (free text) (4) ________________________________________________

Display This Question:

If Did you consult with a veterinary practice/hospital/online vet in the last 12 months (in person o... = Yes

Q4.34 Was this for - (select all that apply)

- Routine health appointment (vaccination, anal gland check, nail clip, medication recheck) (1)
- New health condition/illness (2)
- Advice on euthanasia/end of life care (3)
- Other (4) ________________________________________________

Display This Question:

If Did you consult with a veterinary practice/hospital/online vet in the last 12 months (in person o... = Yes

Q4.35 How many times did your dog go physically to the vet in the last 12 months (or the 12 months before your dog passed away)?

▼ Obtained advice via remote consultation only (email/telephone/online chat) (1) ... I don't know/can't remember (7)

Q4.36 Is/was your dog insured? (Select all that apply)

- Yes (1)
- Yes, but third party only (2)
- No, not insured (3)
- No, not insured, but I have a fund put aside in case of emergencies (4)
- My dog was insured in the past but it became too expensive so I stopped it (5)
- Other (free text) (6) ________________________________________________

Q4.37 Do/did you belong to a health plan at your veterinary practice or online (such as Itchpet.com or Petsathome flea subscription)? (Plans usually consist of a monthly fee, and can include annual vaccinations, preventative care (flea/tick and worms), nurse examination, and sometimes money off medications, blood screening, and/or dental and other procedures). Select all that apply.

- Yes, I belong to a health plan at my veterinary practice (1)
- Yes, I belong to an online flea/tick/worm plan (2)
- No (3)
- No, but I would be interested in joining one (4)
- I don't know (5)

Q4.38 Have you ever attended a senior dog wellness clinic/exam at your veterinary practice (with your vet or veterinary nurse) for this dog? (This is where the veterinary professional focuses on problems specific to ageing and typically performs a complete physical examination, runs tests such as a blood test and/or a urine test to detect early or hidden disease in dogs that appear healthy, or to monitor stable ongoing health problems).

- Yes (1)
- No, my dog is/was not old (2)
- No, my vet does not offer this (3)
- No I have not attended a senior dog wellness clinic/exam, but my vet performs a general physical exam (separately to our annual vaccination appointment) (4)
- Don't know (5)
- Other (6) ________________________________________________

Display This Question:

If Have you ever attended a senior dog wellness clinic/exam at your veterinary practice (with your v... = No, my dog is/was not old

Or Have you ever attended a senior dog wellness clinic/exam at your veterinary practice (with your v... = No, my vet does not offer this

Or Have you ever attended a senior dog wellness clinic/exam at your veterinary practice (with your v... = No I have not attended a senior dog wellness clinic/exam, but my vet performs a general physical exam (separately to our annual vaccination appointment)

Or Have you ever attended a senior dog wellness clinic/exam at your veterinary practice (with your v... = Don't know

Or Or Have you ever attended a senior dog wellness clinic/exam at your veterinary practice (with your v... Other Is Not Empty

Q4.39 Would you be interested in attending one (or attending one when your dog becomes senior if they are under 7 years of age)? Or if your dog has passed away, would you have been interested in attending one?

- Yes (1)
- Yes, but only if it was free (2)
- No (3)
- Don't know (4)
- Other (please state) (5) ________________________________________________

Display This Question:

If Have you ever attended a senior dog wellness clinic/exam at your veterinary practice (with your v... = Yes

Q4.40 Please describe what you remember about your experience at the senior dog wellness clinic/exam. Whether you found this useful, how much did you learn about your dogs health? Would you participate with your dog in another clinic/exam? How often would you do this? Did your vet invite you for the exam, or did you book it yourself?

________________________________________________________________

Q4.41 Would you be willing in the future to fill in a short questionnaire before your next vet visit with this dog or another dog, if sent in advance by email/post or online, or via an app on your mobile? The purpose would be to help you and your vet to pinpoint potential problems, and diagnose them sooner to enable your dog to be in the best health?

- Yes, I already do this at my vet practice. (1)
- Yes, I think this is a good idea, and would be willing to spend time on this. (2)
- Yes, but only if it took less than 10 minutes. (3)
- Yes, but only if it took less than 5 minutes. (4)
- Yes, but only if it took less than 2 minutes. (5)
- No, I would not have time. (6)
- Other (please describe) (7) ________________________________________________

Display This Question:

If Would you be willing in the future to fill in a short questionnaire before your next vet visit wi... = Yes, I think this is a good idea, and would be willing to spend time on this.

Or Would you be willing in the future to fill in a short questionnaire before your next vet visit wi... = Yes, but only if it took less than 10 minutes.

Or Would you be willing in the future to fill in a short questionnaire before your next vet visit wi... = Yes, but only if it took less than 5 minutes.

Or Would you be willing in the future to fill in a short questionnaire before your next vet visit wi... = Yes, but only if it took less than 2 minutes.

Or Would you be willing in the future to fill in a short questionnaire before your next vet visit wi... = Yes, I already do this at my vet practice.

Q4.42 To build up a record of the dog’s health over time, which you would be able to view in simple graphs online/on a mobile app, would you be willing to fill in such a questionnaire -

- Once every few years (1)
- Once a year (2)
- Every 6 months (3)
- Every month (4)
- Other (please describe) (5) ________________________________________________

Display This Question:

If Would you be willing in the future to fill in a short questionnaire before your next vet visit wi... = Yes, I think this is a good idea, and would be willing to spend time on this.

Or Would you be willing in the future to fill in a short questionnaire before your next vet visit wi... = Yes, but only if it took less than 10 minutes.

Or Would you be willing in the future to fill in a short questionnaire before your next vet visit wi... = Yes, but only if it took less than 5 minutes.

Or Would you be willing in the future to fill in a short questionnaire before your next vet visit wi... = Yes, but only if it took less than 2 minutes.

Or Would you be willing in the future to fill in a short questionnaire before your next vet visit wi... = Yes, I already do this at my vet practice.

Q4.43 How would you prefer to fill in such a questionnaire?

- Paper copy sent through the post (1)
- Emailed copy that could be printed out (2)
- Secure internet form that could be filled in online (3)
- Mobile application that could be linked to your vet (4)
- Other (please describe) (5) ________________________________________________

Q4.44 How often do you think a senior dog (7 years plus) should visit a veterinary surgeon if it seems healthy?

- Every 6 months (1)
- Once a year (2)
- Every few years (3)
- Only if they got sick and needed to go (4)
- I don’t know (5)
- If you would like to give us more details on your answer to explain why you think this please write it here in the free text. (6) ________________________________________________

End of Block: Dog Demographics

Start of Block: Deceased dog questions

Display This Question:

If If your oldest dog passed away in the past year, would you be willing to complete the survey for... = Yes – my dog died in the past year and I would be willing to complete the survey for this dog.

Q5.1 Would you mind telling us whether your dog died a natural death or was euthanised?

- My dog died a natural death at home (1)
- My dog died on the way to the vet (2)
- My dog was euthanised at home (3)
- My dog was euthanised at the vet (4)
- Other (please describe) (5) ________________________________________________
- Prefer not to say (6)

Display This Question:

If If your oldest dog passed away in the past year, would you be willing to complete the survey for... = Yes – my dog died in the past year and I would be willing to complete the survey for this dog.

Q5.2 Did you talk to friends/family or use any tools to help you to decide when it was time? (Such as a quality-of-life assessment tool (here is an example - https://journeyspet.com/pet-quality-of-life-scale-calculator/)). Please select all that apply.

- My vet helped me to decide when it was time. (1)
- I was confident that I would know when it was time. (2)
- I needed help from friends and family to help me decide. (3)
- I found resources online that helped me to decide. (4)
- I used quality of life tools to help me decide. (5)
- I joined/belonged to a FB book group/online forum, where I could read about other people’s experiences, & ask questions to help me decide. (6)
- Other (please describe) (7) ________________________________________________

Display This Question:

If Did you talk to friends/family or use any tools to help you to decide when it was time? (Such as... = I used quality of life tools to help me decide.

Q5.3 Did you find the quality-of-life tool useful? Would you recommend it to other dog owners? Is there anything you would add to it, or changes you would make to it to make it easier to use?

________________________________________________________________

Display This Question:

If If your oldest dog passed away in the past year, would you be willing to complete the survey for... = Yes – my dog died in the past year and I would be willing to complete the survey for this dog.

Q5.4 Would you mind telling us about your experience of your dog’s end of life stage? Did you feel supported by your veterinary team through the process?

________________________________________________________________

Display This Question:

If If your oldest dog passed away in the past year, would you be willing to complete the survey for... = Yes – my dog died in the past year and I would be willing to complete the survey for this dog.

Q5.5 Looking back, was there anything you wished they had told you about your dog’s ageing and/or their illness?

________________________________________________________________

Display This Question:

If If your oldest dog passed away in the past year, would you be willing to complete the survey for... = Yes – my dog died in the past year and I would be willing to complete the survey for this dog.

Q5.6 Is there anything you would like other dog owners to know that might help them get through this difficult stage?

________________________________________________________________

End of Block: Deceased dog questions

Start of Block: Dog clinical signs 1

Q6.1 The following questions will describe a specific symptom and ask whether you have ever observed this in the **oldest dog in your household**, or if your oldest dog died in the past year, please complete the questions for that dog, if you are happy to do so.

Q6.2 Did you ever observe the following symptom in your dog? Your dog's teeth appeared stained/brownish with crusty tartar?

- Yes (1)
- No (2)

Display This Question:

If Did you ever observe the following symptom in your dog? Your dog's teeth appeared stained/brownis... = Yes

Q6.3 Did you seek veterinary advice?

- Yes, immediately sought an emergency appointment, (1)
- Yes, on the same day made an appointment for as soon as possible, (2)
- Yes, made an appointment within a week when the condition did not improve, (3)
- Yes, made an appointment within a month when the condition did not improve, (4)
- No, but I mentioned it to the vet when my dog went in for an annual check-up. (5)
- No, I did not contact the vet for this. (6)

Display This Question:

If Did you ever observe the following symptom in your dog? Your dog's teeth appeared stained/brownis... = No

Q6.4 If you had noticed this symptom in your dog, would you have sought veterinary advice?

- Yes, I would have immediately sought an emergency appointment (1)
- Yes, on the same day I would have made an appointment for as soon as possible (2)
- Yes, I would have made an appointment within a week if the condition did not improve (3)
- Yes, I would have made an appointment within a month if the condition did not improve (4)
- No, but I would have mentioned it to the vet when my dog went in for an annual check-up/vaccination. (5)
- No, I would not have contacted the vet for this (6)

Display This Question:

If Did you seek veterinary advice? = No, but I mentioned it to the vet when my dog went in for an annual check-up.

Or Did you seek veterinary advice? = No, I did not contact the vet for this.

Or If you had notice this symptom in your dog, would you have sought veterinary advice? = No, but I would have mentioned it to the vet when my dog went in for an annual check-up/vaccination.

Or If you had notice this symptom in your dog, would you have sought veterinary advice? = No, I would not have contacted the vet for this

Q6.5 Why did you/would you not seek veterinary advice for this symptom? (Tick all that apply)

- This is a normal part of ageing. (1)
- This normally happens occasionally and does not bother the dog or affect their long-term health. (2)
- This is due to my dogs existing medical condition(s) which is being treated with medication. (3)
- I bought/can buy home remedies/tools/treatments for this for my dog online (doesn’t need a prescription). (4)
- I looked/can look for advice online or talk to a knowledgeable friend. (5)
- Covid related issues (emergencies only, don’t want my dog to attend without me present). (6)
- I only go to the vet when it is an emergency, as my dog finds it stressful and/or their behaviour is difficult to manage (fearful/aggressive). (7)
- I find it difficult to get to the vets due to work commitments, and so would only go in an emergency. (8)
- I would not go to a vet as I prefer not to know if my dog has an underlying disease. (9)
- The vet might advise further tests/treatment which I cannot afford at present/or it might increase my insurance premium. (10)
- Other…. (please state). (11) ________________________________________________

Display This Question:

If Did you ever observe the following symptom in your dog? Your dog's teeth appeared stained/brownis... = Yes

Q6.6 How old was your dog when he/she first showed this symptom? (In years)

▼ Under 1 (1) ... 18 (19)

Repeat Questions 6.3 – 6.6 for –

Have you ever observed the following symptom in your dog? - Your dog …..

|  |
| --- |
| - Showed separation anxiety (when left alone will bark/howl, chew/dig/cause destruction, urinate/defecate), |
| - became withdrawn from the family/other pets (e.g., moves to a different room) and/or is reluctant to be petted (dog moves away from you). |
| - Repeatedly licks or chews areas of their body. |
| - became more sensitive to touch (flinches/snaps/vocalises when touched/towel dried) |
| - Needs to be hand fed (can't find the food bowl, or they are refusing to eat). |
| - Needs assistance to drink water (couldn’t find the water bowl, or they refused to drink). |
| - Decreased ability to recognise familiar people/animals inside/outside the house |
| - Forgot training commands and cues they knew previously) |
| - Appears lost or confused (in familiar environment) and/or sometimes stands staring at walls or into space |
| - Can no longer eat hard food (raw hide/crunchy/hard treats etc..) |
| - Breath started to smell bad |
| - Has bouts of vomiting (more than once in a day) |
| - Abdomen has become tense/hard or distended. |
| - Has bouts of diarrhoea and/or mucus/blood in stool |
| - Has slowed down on walks, has less energy |
| - Seems weak after exercise (e.g., lies down). |
| - Has increased panting at rest/laboured breathing, or coughing. |
| - Seems sad/lethargic/depressed/disinterested in life. |
| - Has shown less interest/enthusiasm to go out on walks |
| - Has started to urinate/defecate in the house (when they were previously housetrained successfully)? |
| - Has become incontinent and does not realise when they need to go/goes in own bed |
| - Has smelly/dirty ears, shakes their head, and/or scratched their ears. |
| - Scoots their rear end along the ground or tries to lick or bite their anal area. |
| - has developed sores/hotspots on their skin |
| - Has lumps/swellings in the skin |
| - Changes in the way they walk/run/trot. |
| - Became lame or started to limp/carry a paw. |
| - Has trouble going up/downstairs and/or jumping on the couch. |
| - Has difficulty to get in position to go to the toilet (perhaps because their back legs are weak). |
| - Seems stiff when rising from a nap. |
| - Has shown weakness, occasionally collapse, seem unstable on their feet. |
| - Has suddenly developed a persistent head tilt. |
| - Sometimes drags their foot/feet. |
| - Seemed to suffer a seizure/stroke (uncontrollable shaking/involuntary movements and experiences altered consciousness). |
| - Developed whiteish cloudiness over the centre of one/both their eyes and has difficulty seeing at night? |
| - Has started to bump into things unintentionally, when they didn't do this before. |
| - No longer responds to their name when they did previously. |
| - Spends most of the time sleeping, sleeps more deeply than before. |
| - Wakes you or your family members up at night (whining, crying, barking and/or pacing) |
| - Has shown less interest/enthusiasm to greet you or visitors |
| - Has shown less interest/enthusiasm to play and/or train with you |
| - Has become increasingly grumpy with you/with other dogs, |
| - Drinks a lot more and needs to urinate more |
| - Started to take longer to pee and sometimes strains to pee only a small amount of urine |
| - Has noticeably lost weight/muscle/condition (and the dog is not on a diet?) |
| - Has gained weight (more than normal) |
| - Face seems thin/sunken   Yes  No |

| Page Break |  |
| --- | --- |

End of Block: Dog clinical sign 8

Start of Block: Caregiver burden

Display This Question:

If Please go through the list of conditions below and indicate any that your dog has suffered over t... = Skin problems (skin sores, dry skin, rashes, redness, dandruff, hair loss) [ Diagnosed by a vet ]

Or Please go through the list of conditions below and indicate any that your dog has suffered over t... = Skin problems (skin sores, dry skin, rashes, redness, dandruff, hair loss) [ Diagnosed by someone other than a vet ]

Or Please go through the list of conditions below and indicate any that your dog has suffered over t... = Skin problems (skin sores, dry skin, rashes, redness, dandruff, hair loss) [ Not formally diagnosed, but I feel my dog has it. ]

Or Please go through the list of conditions below and indicate any that your dog has suffered over t... = Kidney/liver disease [ Diagnosed by a vet ]

Or Please go through the list of conditions below and indicate any that your dog has suffered over t... = Kidney/liver disease [ Diagnosed by someone other than a vet ]

Or Please go through the list of conditions below and indicate any that your dog has suffered over t... = Kidney/liver disease [ Not formally diagnosed, but I feel my dog has it. ]

Or Please go through the list of conditions below and indicate any that your dog has suffered over t... = Heart disease (degenerative mitral valve disease (DMVD), dilated cardiomyopathy (DCM), heart murmur, arrhythmias) [ Diagnosed by a vet ]

Or Please go through the list of conditions below and indicate any that your dog has suffered over t... = Heart disease (degenerative mitral valve disease (DMVD), dilated cardiomyopathy (DCM), heart murmur, arrhythmias) [ Diagnosed by someone other than a vet ]

Or Please go through the list of conditions below and indicate any that your dog has suffered over t... = Heart disease (degenerative mitral valve disease (DMVD), dilated cardiomyopathy (DCM), heart murmur, arrhythmias) [ Not formally diagnosed, but I feel my dog has it. ]

Or Please go through the list of conditions below and indicate any that your dog has suffered over t... = Thyroid problems (under/over active) [ Diagnosed by a vet ]

Or Please go through the list of conditions below and indicate any that your dog has suffered over t... = Thyroid problems (under/over active) [ Diagnosed by someone other than a vet ]

Or Please go through the list of conditions below and indicate any that your dog has suffered over t... = Thyroid problems (under/over active) [ Not formally diagnosed, but I feel my dog has it. ]

Or Please go through the list of conditions below and indicate any that your dog has suffered over t... = Epilepsy (fits/seizures) [ Diagnosed by a vet ]

Or Please go through the list of conditions below and indicate any that your dog has suffered over t... = Epilepsy (fits/seizures) [ Diagnosed by someone other than a vet ]

Or Please go through the list of conditions below and indicate any that your dog has suffered over t... = Epilepsy (fits/seizures) [ Not formally diagnosed, but I feel my dog has it. ]

Or Please go through the list of conditions below and indicate any that your dog has suffered over t... = Cancer (Mast cell tumor, melanoma, lymphoma, bone cancer, hemangiosarcoma) [ Diagnosed by a vet ]

Or Please go through the list of conditions below and indicate any that your dog has suffered over t... = Cancer (Mast cell tumor, melanoma, lymphoma, bone cancer, hemangiosarcoma) [ Diagnosed by someone other than a vet ]

Or Please go through the list of conditions below and indicate any that your dog has suffered over t... = Cancer (Mast cell tumor, melanoma, lymphoma, bone cancer, hemangiosarcoma) [ Not formally diagnosed, but I feel my dog has it. ]

Or Please go through the list of conditions below and indicate any that your dog has suffered over t... = Diabetes [ Diagnosed by a vet ]

Or Please go through the list of conditions below and indicate any that your dog has suffered over t... = Diabetes [ Diagnosed by someone other than a vet ]

Or Please go through the list of conditions below and indicate any that your dog has suffered over t... = Diabetes [ Not formally diagnosed, but I feel my dog has it. ]

Or Please go through the list of conditions below and indicate any that your dog has suffered over t... = Gut problems (Inflammatory bowel disease/pancreatitis, colitis) [ Diagnosed by a vet ]

Or Please go through the list of conditions below and indicate any that your dog has suffered over t... = Gut problems (Inflammatory bowel disease/pancreatitis, colitis) [ Diagnosed by someone other than a vet ]

Or Please go through the list of conditions below and indicate any that your dog has suffered over t... = Gut problems (Inflammatory bowel disease/pancreatitis, colitis) [ Not formally diagnosed, but I feel my dog has it. ]

Or Please go through the list of conditions below and indicate any that your dog has suffered over t... = Cushing’s disease, Addison’s disease [ Diagnosed by a vet ]

Or Please go through the list of conditions below and indicate any that your dog has suffered over t... = Cushing’s disease, Addison’s disease [ Diagnosed by someone other than a vet ]

Or Please go through the list of conditions below and indicate any that your dog has suffered over t... = Cushing’s disease, Addison’s disease [ Not formally diagnosed, but I feel my dog has it. ]

Or Please go through the list of conditions below and indicate any that your dog has suffered over t... = Glaucoma [ Diagnosed by a vet ]

Or Please go through the list of conditions below and indicate any that your dog has suffered over t... = Glaucoma [ Diagnosed by someone other than a vet ]

Or Please go through the list of conditions below and indicate any that your dog has suffered over t... = Glaucoma [ Not formally diagnosed, but I feel my dog has it. ]

Or Please go through the list of conditions below and indicate any that your dog has suffered over t... = Loss of eyesight/blind (cataracts (center of lens cloudy or gray), eye removed) [ Diagnosed by a vet ]

Or Please go through the list of conditions below and indicate any that your dog has suffered over t... = Loss of eyesight/blind (cataracts (center of lens cloudy or gray), eye removed) [ Diagnosed by someone other than a vet ]

Or Please go through the list of conditions below and indicate any that your dog has suffered over t... = Loss of eyesight/blind (cataracts (center of lens cloudy or gray), eye removed) [ Not formally diagnosed, but I feel my dog has it. ]

Or Please go through the list of conditions below and indicate any that your dog has suffered over t... = Loss of hearing/deaf [ Diagnosed by a vet ]

Or Please go through the list of conditions below and indicate any that your dog has suffered over t... = Loss of hearing/deaf [ Diagnosed by someone other than a vet ]

Or Please go through the list of conditions below and indicate any that your dog has suffered over t... = Loss of hearing/deaf [ Not formally diagnosed, but I feel my dog has it. ]

Or Please go through the list of conditions below and indicate any that your dog has suffered over t... = Orthopedic problems (fracture/break, Cranial Cruciate Ligament Rupture, hip/elbow dysplasia, osteochondrosis, Arthritis/osteoarthritis) [ Diagnosed by a vet ]

Or Please go through the list of conditions below and indicate any that your dog has suffered over t... = Orthopedic problems (fracture/break, Cranial Cruciate Ligament Rupture, hip/elbow dysplasia, osteochondrosis, Arthritis/osteoarthritis) [ Diagnosed by someone other than a vet ]

Or Please go through the list of conditions below and indicate any that your dog has suffered over t... = Orthopedic problems (fracture/break, Cranial Cruciate Ligament Rupture, hip/elbow dysplasia, osteochondrosis, Arthritis/osteoarthritis) [ Not formally diagnosed, but I feel my dog has it. ]

Or Please go through the list of conditions below and indicate any that your dog has suffered over t... = Spinal problem (Intervertebral Disc Disease (IDD), Spondylosis) [ Diagnosed by a vet ]

Or Please go through the list of conditions below and indicate any that your dog has suffered over t... = Spinal problem (Intervertebral Disc Disease (IDD), Spondylosis) [ Diagnosed by someone other than a vet ]

Or Please go through the list of conditions below and indicate any that your dog has suffered over t... = Spinal problem (Intervertebral Disc Disease (IDD), Spondylosis) [ Not formally diagnosed, but I feel my dog has it. ]

Or Please go through the list of conditions below and indicate any that your dog has suffered over t... = Breathing problems (Brachycephalic Obstructive Airway Syndrome (BOAS)) [ Diagnosed by a vet ]

Or Please go through the list of conditions below and indicate any that your dog has suffered over t... = Breathing problems (Brachycephalic Obstructive Airway Syndrome (BOAS)) [ Diagnosed by someone other than a vet ]

Or Please go through the list of conditions below and indicate any that your dog has suffered over t... = Breathing problems (Brachycephalic Obstructive Airway Syndrome (BOAS)) [ Not formally diagnosed, but I feel my dog has it. ]

Or Please go through the list of conditions below and indicate any that your dog has suffered over t... = Dental disease (including where teeth have been cleaned or removed) [ Diagnosed by a vet ]

Or Please go through the list of conditions below and indicate any that your dog has suffered over t... = Dental disease (including where teeth have been cleaned or removed) [ Diagnosed by someone other than a vet ]

Or Please go through the list of conditions below and indicate any that your dog has suffered over t... = Dental disease (including where teeth have been cleaned or removed) [ Not formally diagnosed, but I feel my dog has it. ]

Or Please go through the list of conditions below and indicate any that your dog has suffered over t... = Doggy dementia (canine cognitive dysfunction) [ Diagnosed by a vet ]

Or Please go through the list of conditions below and indicate any that your dog has suffered over t... = Doggy dementia (canine cognitive dysfunction) [ Diagnosed by someone other than a vet ]

Or Please go through the list of conditions below and indicate any that your dog has suffered over t... = Doggy dementia (canine cognitive dysfunction) [ Not formally diagnosed, but I feel my dog has it. ]

Or Please go through the list of conditions below and indicate any that your dog has suffered over t... = Over-weight/Obese [ Diagnosed by a vet ]

Or Please go through the list of conditions below and indicate any that your dog has suffered over t... = Over-weight/Obese [ Diagnosed by someone other than a vet ]

Or Please go through the list of conditions below and indicate any that your dog has suffered over t... = Over-weight/Obese [ Not formally diagnosed, but I feel my dog has it. ]

Or Please go through the list of conditions below and indicate any that your dog has suffered over t... = Vestibular disease/stroke [ Diagnosed by a vet ]

Or Please go through the list of conditions below and indicate any that your dog has suffered over t... = Vestibular disease/stroke [ Diagnosed by someone other than a vet ]

Or Please go through the list of conditions below and indicate any that your dog has suffered over t... = Vestibular disease/stroke [ Not formally diagnosed, but I feel my dog has it. ]

Or Please go through the list of conditions below and indicate any that your dog has suffered over t... = Other [ Diagnosed by a vet ]

Or Please go through the list of conditions below and indicate any that your dog has suffered over t... = Other [ Diagnosed by someone other than a vet ]

Or Please go through the list of conditions below and indicate any that your dog has suffered over t... = Other [ Not formally diagnosed, but I feel my dog has it. ]

Or If your oldest dog passed away in the past year, would you be willing to complete the survey for... = Yes – my dog died in the past year and I would be willing to complete the survey for this dog.

Q14.1 Please consider each of the following statements and indicate which option most describes how you feel. If your dog has recently passed away, please think about the last three months you spent with your dog.

|  | 0 (Never) (1) | 1 (2) | 2 (3) | 3 (4) | 4 (Nearly always) (5) |
| --- | --- | --- | --- | --- | --- |
| Do you feel that because of the time you spend with your pet that you don't have enough time for yourself? (1) |  |  |  |  |  |
| Do you feel stressed between caring for your pet and trying to meet other responsibilities for your family or work? (2) |  |  |  |  |  |
| Do you feel you have lost control of your life since your pet's illness? (3) |  |  |  |  |  |
| Do you feel angry when you are around your pet? (4) |  |  |  |  |  |
| Do you feel embarrassed over your pet's behaviour? (5) |  |  |  |  |  |
| Do you feel you should be doing more for your pet? (6) |  |  |  |  |  |
| Do you feel you could do a better job in caring for your pet? (7) |  |  |  |  |  |

End of Block: Caregiver burden

Start of Block: Owner demographic

Q15.1 In this section, we will ask you questions about your demographics.

Q15.2 What is your gender?

- Male (1)
- Female (2)
- Other (3)
- Prefer not to say (4)

Q15.3 What is your age category?

- 18 - 29 (1)
- 30 - 39 (2)
- 40 - 49 (3)
- 50 - 59 (4)
- 60 - 69 (5)
- 70 and over (6)
- Prefer not to say (7)

Q15.4 What is your highest level of education?

- University Higher Degree (e.g., MSc, PhD) (1)
- First degree level qualification including foundation degrees, graduate membership of a professional Institute, PGCE (2)
- Diploma in higher education (3)
- Teaching qualification (excluding PGCE) (4)
- Nursing or other medical qualification not yet mentioned. (5)
- A Level (6)
- Welsh Baccalaureate (7)
- International Baccalaureate (8)
- AS Level (9)
- Higher Grade/Advanced Higher (Scotland) (10)
- Certificate of sixth year studies (11)
- GCSE/O Level (12)
- CSE (13)
- Standard/Ordinary (O) Grade / Lower (Scotland) (14)
- Other school (inc. School leaving exam certificate or matriculation) (15)
- None of the above (16)

Q15.5 What is your ethnicity?

- White (includes British, Northern Irish, Irish, Gypsy, Irish Traveller, Roma or any other white background), (1)
- Mixed or Multiple ethnic groups (Includes White and Black Caribbean, White and Black African, White and Asian or any other Mixed or Multiple background). (2)
- Asian or Asian British (includes Indian, Pakistani, Bangladeshi, Chinese or any other Asian background), (3)
- Black, Black British, Caribbean or African (Includes Black British, Caribbean, African or any other Black background), (4)
- Other ethnic group (includes Arab or any other ethnic group), (5)
- Prefer not to say. (6)

Q15.6 What is your level of experience with owning dogs?

- This dog is my first dog ever. (1)
- I had a dog/dogs growing up, but this is my first dog as an adult, (2)
- I had one other dog as an adult, (3)
- I had multiple dogs as an adult, (4)

Q15.7 Do you have experience with dogs in a hobby or professional capacity? (select all that apply)

- Dogs are my hobby (shows, competing in agility, or other dog sports). (1)
- I am a dog breeder/dog trainer/groomer (2)
- I am a dog rescue worker (3)
- I am a vet or vet nurse (4)
- Other main profession that works with dogs (please describe) (5) ________________________________________________
- None of the above. (6)

Q15.8 In which of these brackets does the combined gross (before tax) income of all household members fall?

- Less than £10,000, (1)
- £10,001 – 20,000 (2)
- £20,001 - £30,000, (3)
- £30,001 - £40,000, (4)
- £40,001 - £60,000, (5)
- £60,001 - £80,000, (6)
- More than £80,000 (7)
- Prefer not to say, (8)

Q15.9 Where is your household located? (UK region).

▼ Bath and North East Somerset (1) ... Tyrone (111)

Q15.10 Is there anything else you would like to tell us about the oldest dog in your household?

________________________________________________________________

End of Block: Owner demographic

END

Thank you very much for taking the time to complete this questionnaire.

If you would like to enter our prize draw, please click on the new link below - and enter your name and email address, which will be stored separately to this anonymous survey.

Link to enter prize draw - <https://livpsych.eu.qualtrics.com/jfe/form/SV_8GoVZrbHWtTCs86>

If you feel like you would like more information about your dog’s health, please contact your veterinarian and they can advise you on any health issues your dog may be experiencing.

If you would like to talk to someone about bereavement and loss of a pet, we recommend that you contact the Pet Bereavement Support Service:

The Pet Bereavement Support Service, 0800 096 6606, pbssmail@bluecross.org.uk

Or a qualified Pet Bereavement Counsellor (Member of the British Association for Counsellors and Psychotherapists (MBACP)), <https://www.theralphsite.com/index.php?idPage=22>

In case you need to get in contact with us -

Principle Investigator: Dr Carri Westgarth, Department of Livestock and One Health, University of Liverpool, Leahurst, Chester High Road, Neston, Cheshire, CH64 7TE, Tel: 0151 795 6029, Email: [carri.westgarth@liverpool.ac.uk](mailto:carri.westgarth@liverpool.ac.uk)

Project Researcher: Dr Lisa Wallis, Department of Livestock and One Health, University of Liverpool, Leahurst, Chester High Road, Neston, Cheshire, CH64 7TE, Tel: 0151 795 1426, Email: [lisa.wallis@liverpool.ac.uk](mailto:lisa.wallis@liverpool.ac.uk)

​More information about the project as well as open discussion about the topic of living with senior/geriatric dogs can be found on the project Facebook page ([www.facebook.com/oldagepets](http://www.facebook.com/oldagepets)).

If you would like to be added to the PetSavers mailing list to find out more about the study, please click this link <https://www.surveymonkey.co.uk/r/XTJR7F2>

Supplementary Material should be uploaded separately on submission. Please include any supplementary data, figures and/or tables.

Supplementary material is not typeset so please ensure that all information is clearly presented, the appropriate caption is included in the file and not in the manuscript, and that the style conforms to the rest of the article.
