# Supplementary material for: Cross-sectional United Kingdom surveys demonstrate that owners and veterinary professionals differ in their perceptions of preventive and treatment healthcare needs in ageing dogs
Source: Front Vet Sci. 2024 Apr 4;11:1358480. doi: 10.3389/fvets.2024.1358480 (PMC11024473; doi:10.3389/fvets.2024.1358480)
Supplement: Supplementary file 3 [file Table_3.docx]

Supplementary Material C

# PetSavers Old Age Pets Veterinary Professional Survey

Survey Flow

Block: Introduction and consent (1 Question)

Standard: Screening Questions (2 Questions)

Branch: New Branch

If Do you currently or have you previously (within the past two years) worked as a veterinary profes... No Is Selected

EndSurvey:

Branch: New Branch

If Are you an adult (18 years or over) who lives in the United Kingdom? No Is Selected

EndSurvey:

Standard: Practice information (25 Questions)

Standard: Personal views (16 Questions)

Standard: Clinical questions (7 Questions)

Standard: Clinical questions 2 (6 Questions)

Standard: Demographics (7 Questions)

Start of Block: Introduction and consent

Q1.1 **Informed Consent Form** **PetSavers Old Age Pets Veterinary Professional Survey**

You are being invited to participate in a research study. Before you decide whether to participate, it is important for you to understand why the research is being done and what it will involve. Please take time to read the following information carefully and feel free to ask us if you would like more information or if there is anything that you do not understand.

**1.                What is the purpose of the study?** The purpose of this study is to better understand what senior dog healthcare and treatment advice are currently used by UK veterinary professionals. This includes the offering of senior healthcare plans and clinics, treatment of dogs with chronic and/or mobility problems to reduce pain/improve health, perceived barriers to healthcare seeking for senior dogs, and approaches taken to yearly vaccination appointments. The findings will be used to develop a proforma and guidance tool for use in veterinary consultations with owners of senior dogs.

**2.                Who is involved in running this study?** PetSavers is the funder for this project and Dr Carri Westgarth is the principal investigator. Carri is a Senior Lecturer in Human-Animal Interaction at the University of Liverpool. Dr Lisa Wallis is the principal researcher on the project. This research project has been approved by the University of Liverpool Veterinary Ethics Committee.

**3.                Why should I take part?** We would like veterinary professionals (including veterinary surgeons, veterinary nurses and physiotherapists) from a wide range of different types of services (including private and non-commercial) to complete the survey. You can complete the survey if you currently or recently (within the past two years) conducted consultations on senior dog preventative healthcare/treatment, including carrying out a routine health check of senior/geriatric dogs (such as a yearly booster vaccination appointment or the assessment of senior dogs by a veterinary physiotherapist via referral from a veterinary surgeon).

**4.                Do I have to take part?** No. Participation is entirely voluntary, and participants are free to withdraw at any time during the questionnaire.

**5.                What will happen if I take part?** The survey will take you approximately 20 minutes to complete. Your participation is voluntary and after the first two questions, you are free to omit any of the questions if you wish. You will be asked some general information about your experiences and approaches to treating senior dogs and advising their owners; common physical and behavioural signs that dogs might show; and some general information about yourself (demographics). Your participation is voluntary. You may choose not to participate, and you may withdraw up until the survey is completed. If you decide not to participate or to withdraw from the study, you will not be penalised in any way.
 Your participation remains anonymous. Minimal demographic information will be collected to protect your identity. No personal data will be collected, and all data will be collected and stored safely in compliance with General Data Protection Regulation (GDPR) legislation.

**Contact information**: For further queries, please contact Dr Lisa Wallis, Department of Livestock and One Health, Institute of Infection, Veterinary and Ecological Sciences, University of Liverpool, Leahurst, Chester High Road, Neston, Cheshire, CH64 7TE, Tel: 0151 795 1426, Email: lisa.wallis@liverpool.ac.uk

**Consent statement**:

You understand the purpose of this study, and that you are able to ask questions about it at any time.

You understand that you are free to withdraw your consent for involvement up until reaching the end of the survey.

You understand that if you decide to withdraw from the study, you will not be penalised in any way.

You understand that your name and identifying information will NOT appear in any published document relating to this study, as all information collected in the main survey is completely anonymous, and minimal demographic information is collected to protect your identity.

You understand that the data collected will - though fully anonymised - appear in publications and reports relevant to the purpose of this study and may be used for future ethically approved research.

You understand that your responses will be confidential and remain anonymous.

You understand that no personal data will be collected, and all data will be collected and stored safely in compliance with GDPR legislation.

You understand that you may ask for the results of the study on its completion using the above contact information.

**Please tick 'yes' if you have read and understood the above and give your informed consent to participate in this study.**

- Yes (1)

End of Block: Introduction and consent

Start of Block: Screening Questions

Q2.1 Are you an adult (18 years or over) who lives in the United Kingdom?

- Yes (1)
- No (2)

Q2.2 Do you currently or previously (within the past two years) work as a veterinary professional (veterinary surgeon, veterinary nurse, veterinary physiotherapist (including degree level and postgraduate diploma/MSc in veterinary physiotherapy)).

- Yes (1)
- No (2)

End of Block: Screening Questions

Start of Block: Practice information

Q3.1 In the next section we are going to ask you some questions about your practice.

Q3.2 What is your veterinary-related profession? (Select all that apply)

- Veterinarian (1)
- Veterinary Nurse (2)
- Physiotherapist (3)
- Other animal related (please describe) (4) ________________________________________________

Q3.3 How many years have you practiced as a veterinary-related professional?

- Under 1 year (1)
- 1 - 5 years (2)
- 6 - 10 years (3)
- 11 - 20 years (4)
- Over 20 years (5)

Q3.4 Please indicate the types of practices that you have worked in during the last two years (select all that apply if you work/worked at different types within the last two years).

- I work/worked at more than one practice (1)
- I work/worked as a locum vet/veterinary nurse (2)
- I work/worked as a self employed veterinary physiotherapist (3)
- Clinical practice private corporate (4)
- Clinical practice private independent (5)
- Clinical practice Veterinary school (6)
- Clinical practice charity/trust (7)
- Referral practice/hospital (8)
- University/college or research institute (9)

Q3.5 When answering the following questions, please consider your experiences at your **current (or most recent) practice**, or if you work/worked at **more than one, choose the practice that you feel most affiliated with**. As far as possible, please **answer considering normal working conditions** (rather than what the situation might be during Covid-restricted working conditions).

Q3.6 How many members of staff work at your practice?

- Just me (1)
- 2 - 5 (2)
- 6 - 10 (3)
- More than 10 (4)

Q3.7 What is the name of your practice? Note: This will be kept extremely confidential and only be used to group responses from the same practice so we can more accurately estimate how many practices the responses refer to.

________________________________________________________________

Q3.8 Does your practice consult on senior dog healthcare or treatment (including preventative)?

- Yes (1)
- No (2)
- Other (please describe) (3) ________________________________________________

Q3.9 Does your practice currently carry out a routine health check (regardless of age)?

- Yes (1)
- No (2)
- Not applicable (3)
- Other (4) ________________________________________________

Display This Question:

If What is your veterinary-related profession? (Select all that apply) = Veterinarian

And What is your veterinary-related profession? (Select all that apply) = Veterinary Nurse

Q3.10 Does your practice offer health plans to owners? (Plans usually consist of a monthly fee, and can include annual vaccinations, preventative care (flea/tick and worms), nurse examination, and sometimes money off medications, blood screening, and/or dental and other procedures).

- Yes, we offer at least one health plan at my veterinary practice (1)
- No, and not interested in offering them (2)
- No, but I would be interested in us offering them (3)
- I don't know (4)
- Not applicable (5)

Display This Question:

If Does your practice offer health plans to owners? (Plans usually consist of a monthly fee, and can... = Yes, we offer at least one health plan at my veterinary practice

Q3.11 What type of health plans are offered? (Select all that apply)

- Junior (puppies) (1)
- Adult dogs (2)
- Senior dogs (3)
- Dogs on long-term medication (4)
- Other (please describe) (5) ________________________________________________

Display This Question:

If What type of health plans are offered? (Select all that apply) = Senior dogs

Q3.12 What does your senior healthcare plan include? (Please describe in as much detail as possible).

________________________________________________________________

Display This Question:

If What type of health plans are offered? (Select all that apply) = Senior dogs

Q3.13 How is the senior healthcare plan advertised?

- Advertised in the practice but not offered explicitly to all dogs over a certain age. (1)
- All dogs over a certain age are offered it, for example by sending a letter or asked during a consultation. If so, please state what age dogs are invited to join. (2) ________________________________________________
- Other (please describe) (3) ________________________________________________

Display This Question:

If What is your veterinary-related profession? (Select all that apply) = Veterinarian

And What is your veterinary-related profession? (Select all that apply) = Veterinary Nurse

Q3.14 Does your practice offer senior dog wellness clinic/exams? (Focusing on problems specific to ageing, including a complete physical examination, tests such as a blood test and/or a urine test to detect early or hidden disease in dogs that appear healthy, or to monitor stable ongoing health problems).

- Yes, we offer a senior wellness clinic at my veterinary practice (1)
- Not currently, but we ran one previously (2)
- Not currently, but there was one at another practice I work/worked at (within the last 2 years) (3)
- No, and not interested in offering them (4)
- No, but I would be interested in us offering them (5)
- I don't know (6)
- Not applicable (7)

Display This Question:

If Does your practice offer senior dog wellness clinic/exams? (Focusing on problems specific to agei... = Yes, we offer a senior wellness clinic at my veterinary practice

Q3.15 Who runs your senior wellness clinic?

- Myself (1)
- A veterinary surgeon colleague (2)
- A veterinary nurse (3)
- Other (4) ________________________________________________

Display This Question:

If Does your practice offer senior dog wellness clinic/exams? (Focusing on problems specific to agei... = Yes, we offer a senior wellness clinic at my veterinary practice

Q3.16 Please describe what the senior dog wellness clinics/exams include (e.g. physical exam, diagnostics and preventative advice (including diet, dental care, exercise, and modification of home environment)) and your views on whether you think each of the components are useful.

________________________________________________________________

Display This Question:

If Does your practice offer senior dog wellness clinic/exams? (Focusing on problems specific to agei... = Not currently, but we ran one previously

Q3.17 Why does your practice no longer run them? (Select all that apply, please take into account your normal working conditions rather than what the situation might be during Covid-restricted working conditions).

- Very poor client uptake (1)
- Lack of time to run them (2)
- The person who was responsible for them left (3)
- Lack of space/rooms to run the clinic (4)
- Lack of personnel to run them (5)
- Other (please describe) (6) ________________________________________________
- Don't know (7)

Display This Question:

If Does your practice offer senior dog wellness clinic/exams? (Focusing on problems specific to agei... = No, and not interested in offering them

And Does your practice offer senior dog wellness clinic/exams? (Focusing on problems specific to agei... = No, but I would be interested in us offering them

Q3.18 Are there any particular reasons why your practice does not offer specific senior health clinics or consultations? (Select all that apply, please take into account your normal working conditions rather than what the situation might be during Covid-restricted working conditions).

- I think client uptake would be poor due to the cost of diagnostic tests (1)
- We do not have time to run extra clinics (2)
- No one is interested in running them (3)
- We don't have the space/room to run them (4)
- We cannot spare personnel to run them (5)
- At some point in the future we hope to offer them (6)
- Other (please describe) (7) ________________________________________________
- Don't know (8)

End of Block: Practice information

Start of Block: Personal views

Q4.1 In the next section we are going to ask you some questions about your opinion on senior dog healthcare.

Q4.2 At what age would you consider a medium sized dog (such as an English Cocker Spaniel) to be ‘senior’? (In years)

▼ 4 (1) ... 12 (9)

Q4.3 Do you think senior dogs require a different consultation approach than with younger dogs?

- Yes, and I already do this. Please describe why. (1) ________________________________________________
- Yes, but I don’t currently do this. Please describe why. (2) ________________________________________________
- No, all consultations should follow the same procedure regardless of age. Please describe why. (3) ________________________________________________
- Other (please describe) (4) ________________________________________________

Q4.4 How often do you think a senior dog should visit a veterinary surgeon if they seem healthy?

- Every 6 months. Please describe why. (1) ________________________________________________
- Once a year. Please describe why. (2) ________________________________________________
- Every few years. Please describe why. (3) ________________________________________________
- Only if they got sick and needed to go. Please describe why. (4) ________________________________________________
- I don't know (5)

Display This Question:

If What is your veterinary-related profession? (Select all that apply) = Veterinarian

And What is your veterinary-related profession? (Select all that apply) = Veterinary Nurse

Q4.5 Do you personally believe that senior and geriatric dogs should continue to receive annual booster vaccinations?

- Yes (1)
- No (2)
- It depends (please describe) (3) ________________________________________________

Display This Question:

If What is your veterinary-related profession? (Select all that apply) = Veterinarian

And What is your veterinary-related profession? (Select all that apply) = Veterinary Nurse

Q4.6 In your experience, in what situations and for what reasons would you advise not to give an annual booster vaccination to a senior/geriatric dog?

________________________________________________________________

Display This Question:

If Do you personally believe that senior and geriatric dogs should continue to receive annual booste... = No

And Do you personally believe that senior and geriatric dogs should continue to receive annual booste... = It depends (please describe)

Q4.7 At what age might you advise to stop vaccinating geriatric dogs?

________________________________________________________________

Q4.8 Do you use quality of life tools with your owners when considering end-of-life care? (here is an example - https://journeyspet.com/pet-quality-of-life-scale-calculator/)).

- No (1)
- Yes, I give one to each owner (2)
- Yes, I tell owners to look for them online (3)
- Not explicitly, but I will discuss them with owners if they bring it up. (4)
- Other (please describe) (5) ________________________________________________

Display This Question:

If Do you use quality of life tools with your owners when considering end-of-life care? (here is an... = Yes, I give one to each owner

And Do you use quality of life tools with your owners when considering end-of-life care? (here is an... = Yes, I tell owners to look for them online

And Do you use quality of life tools with your owners when considering end-of-life care? (here is an... = Not explicitly, but I will discuss them with owners if they bring it up.

Q4.9 Which quality of life tools do you recommend?

________________________________________________________________

Display This Question:

If Do you use quality of life tools with your owners when considering end-of-life care? (here is an... = No

Q4.10 Why do you not use quality of life tools? (Please select all that are applicable).

- I haven't found one yet that I find useful. (1)
- Most owners have already made decision to euthanise before arriving. (2)
- Other (please describe) (3) ________________________________________________

Display This Question:

If Do you use quality of life tools with your owners when considering end-of-life care? (here is an... = Yes, I give one to each owner

And Do you use quality of life tools with your owners when considering end-of-life care? (here is an... = Yes, I tell owners to look for them online

And Do you use quality of life tools with your owners when considering end-of-life care? (here is an... = Not explicitly, but I will discuss them with owners if they bring it up.

Q4.11 Do you find current quality-of-life tools useful?

- Yes (If so, why?) (1) ________________________________________________
- No (If so, why?) (2) ________________________________________________

Display This Question:

If Do you use quality of life tools with your owners when considering end-of-life care? (here is an... = Yes, I give one to each owner

And Do you use quality of life tools with your owners when considering end-of-life care? (here is an... = Yes, I tell owners to look for them online

And Do you use quality of life tools with your owners when considering end-of-life care? (here is an... = Not explicitly, but I will discuss them with owners if they bring it up.

Q4.12 Is there anything you would add to them, or changes you would make to it to make them easier to use?

________________________________________________________________

Q4.13 Is there anything in particular that you have noticed that you wish dog owners knew that might help them get through this difficult end-of-life stage?

________________________________________________________________

Q4.14 Do you think it would be a good idea to ask owners of senior dogs to fill in a short health questionnaire before their visit, for example if sent in advance by email/post or online, or via a mobile app? The purpose would be to help you and the owners to pinpoint potential problems, and diagnose them sooner to enable dogs to be in the best health.

- Yes, we already do this at my practice. (1)
- Yes, I think this is a good idea, and would be willing to spend time on this in my consultations. (2)
- Yes, but only if the admin team were responsible for flagging up the potential problems. (3)
- Yes, but only if the analysis of the questionnaire was automated, and any potential problems were flagged by our computer system. (4)
- No, I would not have time to add this tool to my already overloaded schedule in consultations. (5)
- Other (please describe) (6) ________________________________________________

Display This Question:

If Do you think it would be a good idea to ask owners of senior dogs to fill in a short health quest... = Yes, we already do this at my practice.

And Do you think it would be a good idea to ask owners of senior dogs to fill in a short health quest... = Yes, I think this is a good idea, and would be willing to spend time on this in my consultations.

And Do you think it would be a good idea to ask owners of senior dogs to fill in a short health quest... = Yes, but only if the admin team were responsible for flagging up the potential problems.

And Do you think it would be a good idea to ask owners of senior dogs to fill in a short health quest... = Yes, but only if the analysis of the questionnaire was automated, and any potential problems were flagged by our computer system.

Q4.15 To build up a record of the dog’s health over time, which would be viewable in simple graphs online/on a mobile app, how often do you think owners should fill in a questionnaire?

- Once every few years (1)
- Once a year (2)
- Every 6 months (3)
- Every month (4)
- Other (please describe) (5) ________________________________________________

Display This Question:

If Do you think it would be a good idea to ask owners of senior dogs to fill in a short health quest... = Yes, we already do this at my practice.

And Do you think it would be a good idea to ask owners of senior dogs to fill in a short health quest... = Yes, I think this is a good idea, and would be willing to spend time on this in my consultations.

And Do you think it would be a good idea to ask owners of senior dogs to fill in a short health quest... = Yes, but only if the admin team were responsible for flagging up the potential problems.

And Do you think it would be a good idea to ask owners of senior dogs to fill in a short health quest... = Yes, but only if the analysis of the questionnaire was automated, and any potential problems were flagged by our computer system.

Q4.16 How would you prefer owners to fill in such a questionnaire? (Please select all that apply).

- Paper copy sent through the post (1)
- Emailed copy that could be printed out (2)
- Secure internet form that could be filled in online (3)
- Mobile application linked to your practice (4)
- Other (please describe) (5) ________________________________________________

Display This Question:

If Do you think it would be a good idea to ask owners of senior dogs to fill in a short health quest... = No, I would not have time to add this tool to my already overloaded schedule in consultations.

Q4.17 Why do you think that owner questionnaires are not a good idea?

________________________________________________________________

End of Block: Personal views

Start of Block: Clinical questions

Q5.1 In the next section we are going to ask you some questions about clinical signs in senior dogs.

Q5.2 How important do you think it is for owners to seek veterinary advice for their senior dogs if they display each symptom listed below?

|  | Extremely important (1) | Very important (2) | Moderately important (3) | Neutral (4) | Slightly important (5) | Low importance (6) | Not at all important (7) |
| --- | --- | --- | --- | --- | --- | --- | --- |
| teeth appear stained/brownish with crusty tartar (1) |  |  |  |  |  |  |  |
| breath smells bad (2) |  |  |  |  |  |  |  |
| drinks a lot more and needs to urinate more (3) |  |  |  |  |  |  |  |
| became withdrawn from the family/pets (moves to a different room), and/or is reluctant to be petted? (4) |  |  |  |  |  |  |  |
| urinates/defecates in the house (when they were previously housetrained) (5) |  |  |  |  |  |  |  |
| face seems thin/sunken (6) |  |  |  |  |  |  |  |
| gained weight (more than normal) (7) |  |  |  |  |  |  |  |
| has difficulty to get in position to go to the toilet (because back legs are weak) (8) |  |  |  |  |  |  |  |

Q5.3 How important do you think it is for owners to seek veterinary advice for their senior dogs if they display each symptom listed below?

|  | Extremely important (1) | Very important (2) | Moderately important (3) | Neutral (4) | Slightly important (5) | Low importance (6) | Not at all important (7) |
| --- | --- | --- | --- | --- | --- | --- | --- |
| sores/hotspots on their skin (1) |  |  |  |  |  |  |  |
| sad/lethargic/ depressed/ disinterested in life (2) |  |  |  |  |  |  |  |
| incontinent (does not realise when they need to go) goes in own bed (3) |  |  |  |  |  |  |  |
| needs assistance to drink water (can't find the water bowl, or they refused to drink) (4) |  |  |  |  |  |  |  |
| can no longer eat hard food (raw hide/ crunchy /hard treats etc..) (5) |  |  |  |  |  |  |  |
| bumps into things unintentionally, when they didn't do this before (6) |  |  |  |  |  |  |  |
| decreased ability to recognise familiar people/animals inside/outside the house (7) |  |  |  |  |  |  |  |
| abdomen became tense/hard or distended (8) |  |  |  |  |  |  |  |

Q5.4 How important do you think it is for owners to seek veterinary advice for their senior dogs if they display each symptom listed below?

|  | Extremely important (1) | Very important (2) | Moderately important (3) | Neutral (4) | | Slightly important (5) | Low importance (6) | Not at all important (7) |
| --- | --- | --- | --- | --- | --- | --- | --- | --- |
| slowed down on walks, and has less energy (1) |  |  |  |  |  | |  |  |
| smelly/dirty ears, shakes head, and/or scratches ears (2) |  |  |  |  |  | |  |  |
| gait has changed (walk/trot/run) (3) |  |  |  |  |  | |  |  |
| bouts of diarrhoea and/or mucus/blood in their stool (4) |  |  |  |  |  | |  |  |
| takes longer to pee and sometimes strains to pee only a small amount of urine (5) |  |  |  |  |  | |  |  |
| scoots their rear end along the ground or tries to lick/bite their anal area (6) |  |  |  |  |  | |  |  |
| develops a persistent head tilt (7) |  |  |  |  |  | |  |  |
| increased panting at rest/laboured breathing, or coughing (8) |  |  |  |  |  | |  |  |

Q5.5 How important do you think it is for owners to seek veterinary advice for their senior dogs if they display each symptom listed below?

|  | Extremely important (1) | Very important (2) | Moderately important (3) | Neutral (4) | Slightly important (5) | Low importance (6) | Not at all important (7) |
| --- | --- | --- | --- | --- | --- | --- | --- |
| spends nearly all of their time sleeping, and sleeps more deeply than before (1) |  |  |  |  |  |  |  |
| wakes owner up at night (whining, crying, barking and/or pacing) (2) |  |  |  |  |  |  |  |
| developed whiteish cloudiness over the center of one/both their eyes and has difficulty seeing at night (3) |  |  |  |  |  |  |  |
| repeatedly licks or chews areas of their body (4) |  |  |  |  |  |  |  |
| weak after exercise (e.g., lies down when they stop) (5) |  |  |  |  |  |  |  |
| no longer responds to their name when they did previously (6) |  |  |  |  |  |  |  |
| stiff when rising after a nap (7) |  |  |  |  |  |  |  |
| shows less interest/enthusiasm to go out on walks (8) |  |  |  |  |  |  |  |

Q5.6 How important do you think it is for owners to seek veterinary advice for their senior dogs if they display each symptom listed below?

|  | Extremely important (1) | Very important (2) | Moderately important (3) | Neutral (4) | Slightly important (5) | Low importance (6) | Not at all important (7) |
| --- | --- | --- | --- | --- | --- | --- | --- |
| needs to be hand fed (can't find the food bowl, or refuses to eat) (1) |  |  |  |  |  |  |  |
| forgets training commands and cues they knew previously (2) |  |  |  |  |  |  |  |
| developed separation anxiety (when left alone will bark/howl, chew/dig/cause destruction, urinate/defecate) (3) |  |  |  |  |  |  |  |
| has trouble going up/downstairs and/or jumping on the couch (4) |  |  |  |  |  |  |  |
| shows weakness, occasional collapse, unstable on their feet (5) |  |  |  |  |  |  |  |
| more sensitive to touch (flinches/snaps/vocalises when touched/towel dried) (6) |  |  |  |  |  |  |  |
| shows less interest/enthusiasm to greet owner/visitors (7) |  |  |  |  |  |  |  |
| noticeably lost weight/muscle/condition (and dog not on a diet) (8) |  |  |  |  |  |  |  |

Q5.7 How important do you think it is for owners to seek veterinary advice for their senior dogs if they display each symptom listed below?

|  | Extremely important (1) | Very important (2) | Moderately important (3) | Neutral (4) | Slightly important (5) | Low importance (6) | Not at all important (7) |
| --- | --- | --- | --- | --- | --- | --- | --- |
| appears lost or confused (in a familiar environment) and/or sometimes stands staring at walls/into space (1) |  |  |  |  |  |  |  |
| lumps/swellings in their skin (2) |  |  |  |  |  |  |  |
| seizure/stroke (uncontrollable shaking/involuntary movements and/or experienced altered consciousness) (3) |  |  |  |  |  |  |  |
| sometimes drags their foot/feet (4) |  |  |  |  |  |  |  |
| became lame, or started to limp/carry a paw (5) |  |  |  |  |  |  |  |
| bouts of vomiting (more than once in a day) (6) |  |  |  |  |  |  |  |
| shows less interest/enthusiasm to play and/or train with owner than they would normally (7) |  |  |  |  |  |  |  |
| became increasingly grumpy with owner/other dogs (8) |  |  |  |  |  |  |  |

|  |  |
| --- | --- |

Q6.1 How often do you think owners attribute the following symptoms to ‘just old age’ and do not seek veterinary advice for their senior dog/s?

|  | All the time (1) | Usually, in about 90% of cases (2) | Frequently, in about 70% of cases (3) | Sometimes, in about 50% of cases (4) | Occasionally, in about 30% of cases (5) | Rarely, in less than 10% of cases (6) | Never (7) |
| --- | --- | --- | --- | --- | --- | --- | --- |
| teeth appear stained/brownish with crusty tartar (1) |  |  |  |  |  |  |  |
| breath smells bad (2) |  |  |  |  |  |  |  |
| drinks a lot more and needs to urinate more (3) |  |  |  |  |  |  |  |
| became withdrawn from the family/pets (moves to a different room), and/or is reluctant to be petted? (4) |  |  |  |  |  |  |  |
| urinates/defecates in the house (when they were previously housetrained) (5) |  |  |  |  |  |  |  |
| face seems thin/sunken (6) |  |  |  |  |  |  |  |
| gained weight (more than normal) (7) |  |  |  |  |  |  |  |
| has difficulty to get in position to go to the toilet (because back legs are weak) (8) |  |  |  |  |  |  |  |

Q6.2 How often do you think owners attribute the following symptoms to ‘just old age’ and do not seek veterinary advice for their senior dog/s?

|  | All the time (1) | Usually, in about 90% of cases (2) | Frequently, in about 70% of cases (3) | Sometimes, in about 50% of cases (4) | Occasionally, in about 30% of cases (5) | Rarely, in less than 10% of cases (6) | Never (7) |
| --- | --- | --- | --- | --- | --- | --- | --- |
| sores/hotspots on their skin (1) |  |  |  |  |  |  |  |
| sad/lethargic /depressed/ disinterested in life (2) |  |  |  |  |  |  |  |
| incontinent (does not realise when they need to go) goes in own bed (3) |  |  |  |  |  |  |  |
| needs assistance to drink water (can't find the water bowl, or they refused to drink) (4) |  |  |  |  |  |  |  |
| can no longer eat hard food (raw hide/crunchy/hard treats etc..) (5) |  |  |  |  |  |  |  |
| bumps into things unintentionally, when they didn't do this before (6) |  |  |  |  |  |  |  |
| decreased ability to recognise familiar people/animals inside/outside the house (7) |  |  |  |  |  |  |  |
| abdomen became tense/hard or distended (8) |  |  |  |  |  |  |  |

|  |
| --- |

Q6.3 How often do you think owners attribute the following symptoms to ‘just old age’ and do not seek veterinary advice for their senior dog/s?

|  | All the time (1) | | Usually, in about 90% of cases (2) | Frequently, in about 70% of cases (3) | Sometimes, in about 50% of cases (4) | Occasionally, in about 30% of cases (5) | Rarely, in less than 10% of cases (6) | Never (7) |
| --- | --- | --- | --- | --- | --- | --- | --- | --- |
| slowed down on walks, and has less energy (1) |  |  | |  |  |  |  |  |
| smelly/dirty ears, shakes head, and/or scratches ears (2) |  |  | |  |  |  |  |  |
| gait has changed (walk/trot/run) (3) |  |  | |  |  |  |  |  |
| bouts of diarrhoea and/or mucus/blood in their stool (4) |  |  | |  |  |  |  |  |
| takes longer to pee and sometimes strains to pee only a small amount (5) |  |  | |  |  |  |  |  |
| scoots their rear end along the ground or tries to lick/bite their anal area (6) |  |  | |  |  |  |  |  |
| develops a persistent head tilt (7) |  |  | |  |  |  |  |  |
| increased panting at rest/laboured breathing, or coughing (8) |  |  | |  |  |  |  |  |

Q6.4 How often do you think owners attribute the following symptoms to ‘just old age’ and do not seek veterinary advice for their senior dog/s?

|  | All the time (1) | Usually, in about 90% of cases (2) | Frequently, in about 70% of cases (3) | Sometimes, in about 50% of cases (4) | Occasionally, in about 30% of cases (5) | Rarely, in less than 10% of cases (6) | Never (7) |
| --- | --- | --- | --- | --- | --- | --- | --- |
| spends nearly all of their time sleeping, and sleeps more deeply than before (1) |  |  |  |  |  |  |  |
| wakes owner up at night (whining, crying, barking and/or pacing) (2) |  |  |  |  |  |  |  |
| developed whiteish cloudiness over the center of one/both their eyes and has difficulty seeing at night (3) |  |  |  |  |  |  |  |
| repeatedly licks or chews areas of their body (4) |  |  |  |  |  |  |  |
| weak after exercise (e.g., lies down when they stop) (5) |  |  |  |  |  |  |  |
| no longer responds to their name when they did previously (6) |  |  |  |  |  |  |  |
| stiff when rising after a nap (7) |  |  |  |  |  |  |  |
| shows less interest/enthusiasm to go out on walks (8) |  |  |  |  |  |  |  |

Q6.5 How often do you think owners attribute the following symptoms to ‘just old age’ and do not seek veterinary advice for their senior dog/s?

|  | All the time (1) | Usually, in about 90% of cases (2) | Frequently, in about 70% of cases (3) | Sometimes, in about 50% of cases (4) | Occasionally, in about 30% of cases (5) | Rarely, in less than 10% of cases (6) | Never (7) |
| --- | --- | --- | --- | --- | --- | --- | --- |
| needs to be hand fed (can't find the food bowl, or refuses to eat) (1) |  |  |  |  |  |  |  |
| forgets training commands and cues they knew previously (2) |  |  |  |  |  |  |  |
| developed separation anxiety (when left alone will bark/howl, chew/dig/cause destruction, urinate/defecate) (3) |  |  |  |  |  |  |  |
| has trouble going up/downstairs and/or jumping on the couch (4) |  |  |  |  |  |  |  |
| shows weakness, occasional collapse, unstable on their feet (5) |  |  |  |  |  |  |  |
| more sensitive to touch (flinches/snaps/vocalises when touched/towel dried) (6) |  |  |  |  |  |  |  |
| shows less interest/enthusiasm to greet owner/visitors (7) |  |  |  |  |  |  |  |
| noticeably lost weight/muscle/condition (and dog not on a diet) (8) |  |  |  |  |  |  |  |

Q6.6 How often do you think owners attribute the following symptoms to ‘just old age’ and do not seek veterinary advice for their senior dog/s?

|  | All the time (1) | Usually, in about 90% of cases (2) | Frequently, in about 70% of cases (3) | Sometimes, in about 50% of cases (4) | Occasionally, in about 30% of cases (5) | Rarely, in less than 10% of cases (6) | Never (7) |
| --- | --- | --- | --- | --- | --- | --- | --- |
| appears lost or confused (in a familiar environment) and/or sometimes stands staring at walls/into space (1) |  |  |  |  |  |  |  |
| lumps/swellings in their skin (2) |  |  |  |  |  |  |  |
| seizure/stroke (uncontrollable shaking/involuntary movements and/or experienced altered consciousness) (3) |  |  |  |  |  |  |  |
| sometimes drags their foot/feet (4) |  |  |  |  |  |  |  |
| became lame, or started to limp/carry a paw (5) |  |  |  |  |  |  |  |
| bouts of vomiting (more than once in a day) (6) |  |  |  |  |  |  |  |
| shows less interest/enthusiasm to play and/or train with owner than they would normally (7) |  |  |  |  |  |  |  |
| became increasingly grumpy with owner/other dogs (8) |  |  |  |  |  |  |  |

End of Block: Clinical questions

Start of Block: Demographics

Q7.1 In the next section we are going to ask you some demographic questions.

Q7.2 Please indicate your age category

- less than 30 (1)
- 30 - 40 (2)
- 41 - 50 (3)
- 51 - 60 (4)
- 61 - 70 (5)
- >70 (6)

Q7.3 What is your gender?

- Male (1)
- Female (2)
- Non-binary / third gender (3)
- Prefer not to say (4)

Q7.4 What is your highest level of education?

- University Higher Degree (e.g., MSc, PhD) (1)
- First degree level qualification including foundation degrees, graduate membership of a professional Institute, PGCE (2)
- Diploma in higher education (3)
- Teaching qualification (excluding PGCE) (4)
- Nursing or other medical qualification not yet mentioned. (5)
- A Level (6)
- Welsh Baccalaureate (7)
- International Baccalaureate (8)
- AS Level (9)
- Higher Grade/Advanced Higher (Scotland) (10)
- Certificate of sixth year studies (11)
- GCSE/O Level (12)

Q7.5 What is your ethnicity?

- White (includes British, Northern Irish, Irish, Gypsy, Irish Traveller, Roma or any other white background), (1)
- Mixed or Multiple ethnic groups (Includes White and Black Caribbean, White and Black African, White and Asian or any other Mixed or Multiple background). (2)
- Asian or Asian British (includes Indian, Pakistani, Bangladeshi, Chinese or any other Asian background), (3)
- Black, Black British, Caribbean or African (Includes Black British, Caribbean, African or any other Black background), (4)
- Other ethnic group (includes Arab or any other ethnic group), (5)
- Prefer not to say. (6)

Q7.6 In which of these brackets does the combined gross (before tax) income of all household members fall?

- Less than £10,000, (1)
- £10,001 – 20,000 (2)
- £20,001 - £30,000, (3)
- £30,001 - £40,000, (4)
- £40,001 - £60,000, (5)
- £60,001 - £80,000, (6)
- More than £80,000 (7)
- Prefer not to say, (8)

Q7.7 In which region of the UK do you practice?

▼ Bath and North East Somerset (1) ... Tyrone (111)

End of Block: Demographics

End of Survey

**Thank you very much for taking the time to complete this questionnaire.**
If you would like to enter our **prize draw**, please click on the link below - and enter your name and email address (which will not be linked to your survey responses). Entrants will be informed of the result of the prize draw by email. 
Link to enter prize draw - <https://livpsych.eu.qualtrics.com/jfe/form/SV_8GoVZrbHWtTCs86>

If any of the topics within the survey around management of older animals have raised any concerns for you then Vetlife is a good source of further discussion, support and advice. Call the vetlife helpline on 0303 040 2551, or send a confidential message via the vetlife website <https://helpline.vetlife.org.uk/>

If you would like to talk to someone about the difficulties in working in senior dog care and animal euthanasia, we recommend that you contact the Pet Bereavement Support Service:
The Pet Bereavement Support Service
0800 096 6606, [pbssmail@bluecross.org.uk](mailto:pbssmail@bluecross.org.uk)
or a qualified Pet Bereavement Counsellor (Member of the British Association for Counsellors and Psychotherapists (MBACP)), <https://www.theralphsite.com/index.php?idPage=22>

In case you need to get in contact with us -
Principle Investigator: Dr Carri Westgarth, Department of Livestock and One Health, University of Liverpool, Leahurst, Chester High Road, Neston, Cheshire, CH64 7TE, Tel: 0151 795 6029, Email: [carri.westgarth@liverpool.ac.uk](mailto:carri.westgarth@liverpool.ac.uk)

Project Researcher: Dr Lisa Wallis, Department of Livestock and One Health, University of Liverpool, Leahurst, Chester High Road, Neston, Cheshire, CH64 7TE, Tel: 0151 795 1426, Email: [lisa.wallis@liverpool.ac.uk](mailto:lisa.wallis@liverpool.ac.uk)

​More information about the project as well as open discussion about the topic of living with senior/geriatric dogs can be found on the project Facebook page ([www.facebook.com/oldagepets](http://www.facebook.com/oldagepets)).
